# Cu-catalyzed, Mn-mediated propargylation and allenylation of aldehydes with propargyl bromides

**DOI:** 10.1186/s13065-022-00803-3

**Published:** 2022-03-18

**Authors:** Rongli Zhang, Yanping Xia, Yuchen Yan, Lu Ouyang

**Affiliations:** 1 Xuzhou Medical University, Tongshan Road 209, Xuzhou, 221004 China; 2 School of Pharmaceutical Sciences, Gannan Medical University, Ganzhou, 341000 China

**Keywords:** Propargylation, Allenylation, Mn powder, Cu-catalyzed, Gram scale

## Abstract

A simple, practical, and high chemo-selective method for the synthesis of propargyl alcohol and allenyl alcohols via Cu-catalyzed, Mn-mediated propargylation and allenylation of aldehydes with propargyl bromides has been established. When 3-bromo-1-propyne was conducted under the standard condition, the aldehydes were transformed to the corresponding propargylation products completely, while when 1-bromo-2-pentyne was used, allenic alcohol was the only product. Variety of homopropargyl alcohols and allenyl alcohols were obtained in high yields and the reaction is compatible with broad substrate scopes. In addition, the large-scale reaction could also be proceeded smoothly indicating the potential synthetic applications of this transformation.

**Graphical Abstract:**

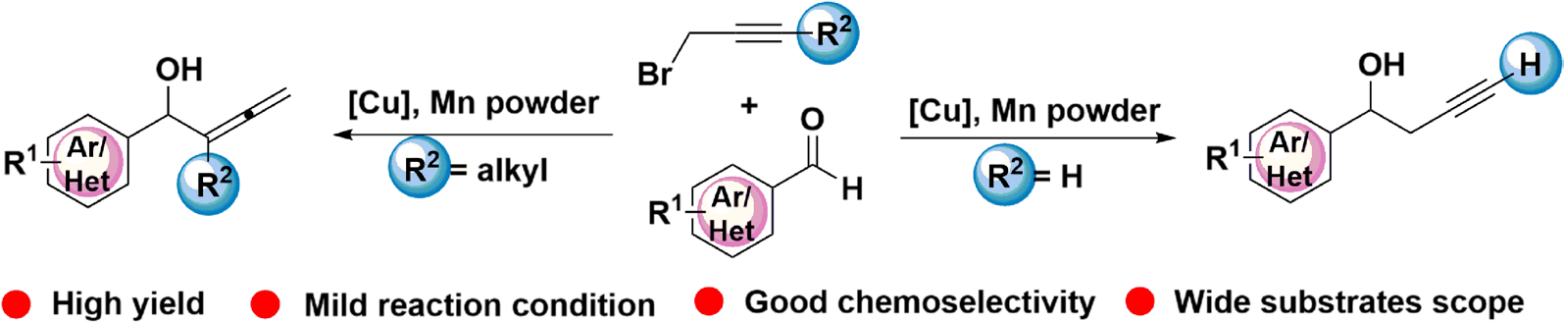

**Supplementary Information:**

The online version contains supplementary material available at 10.1186/s13065-022-00803-3.

## Introduction

Propargyl and allenyl groups are not only valuable building blocks for further manipulations and organic transformations in organic synthesis [1–7], but also sever as active structural moieties in plentiful functional molecules which are important in bioactive molecules, pharmaceuticals agents and natural products [8–13]. Thus, this interesting and promising synthetic method has been attracting a great deal of attentions [14–23]. Numerous methods have been established by using propargyl halides and metals to produce the nucleophilic character of the propargyl metal species [24–26]. When the nucleophilic receptor is an aldehyde, the homopropargyl alcohol can be obtained by the nucleophilic addition of propargyl metal species and aldehyde [27, 28]. Variety of metals, including In [29–31], Sb [32], Pb [33], Ti [34], Cr [35], Ga [36], Sn [37], Zn [38, 39], Mn [40] and Sc [41], have been used for this coupling reaction which could afford the corresponding homopropargyl alcohols. While, the by-product allenyl alcohol is inevitable, which can be owned to the rearrangement of the crucial intermediate progargyl metal species to allenyl metal species [42]. Therefore, a mixture of homopropargyl alcohol and allenyl alcohol were generally obtained. Despite the encouraging progress has been made [43–45], long reaction time–cost, moderate yields and low chemo-selectivity has limited the applications. Therefore, there is still demands for the improved method with respect to selectivities for homopropargyl alcohol and allenyl alcohols.

As we known, Cu catalyst, is not only abundant, easy to utilize, and relatively insensitive to water and air, but also has advantageous for the controllable access to Cu(0), Cu(I), Cu(II), and Cu(III) oxidation states [46, 47]; possibly because of its single-electron transfer (SET) and two-electron processes (TEPs) pathway [48, 49]; which make the catalytic system with high catalytic activities and rate. Moreover, Manganese has been widely used in organic reactions by virtue of its environmentally benign and sustainable nature, low cost and versatile reactivity [50, 51]. Up to now, there were only few examples had been reported, but they showed the activity of Mn in the proparylation reaction. So will the combination of Cu-catalyst and Mn powder increase the catalytic efficiency in the proparylation of propargyl bromide with aldehyde?

**Fig. 1:**
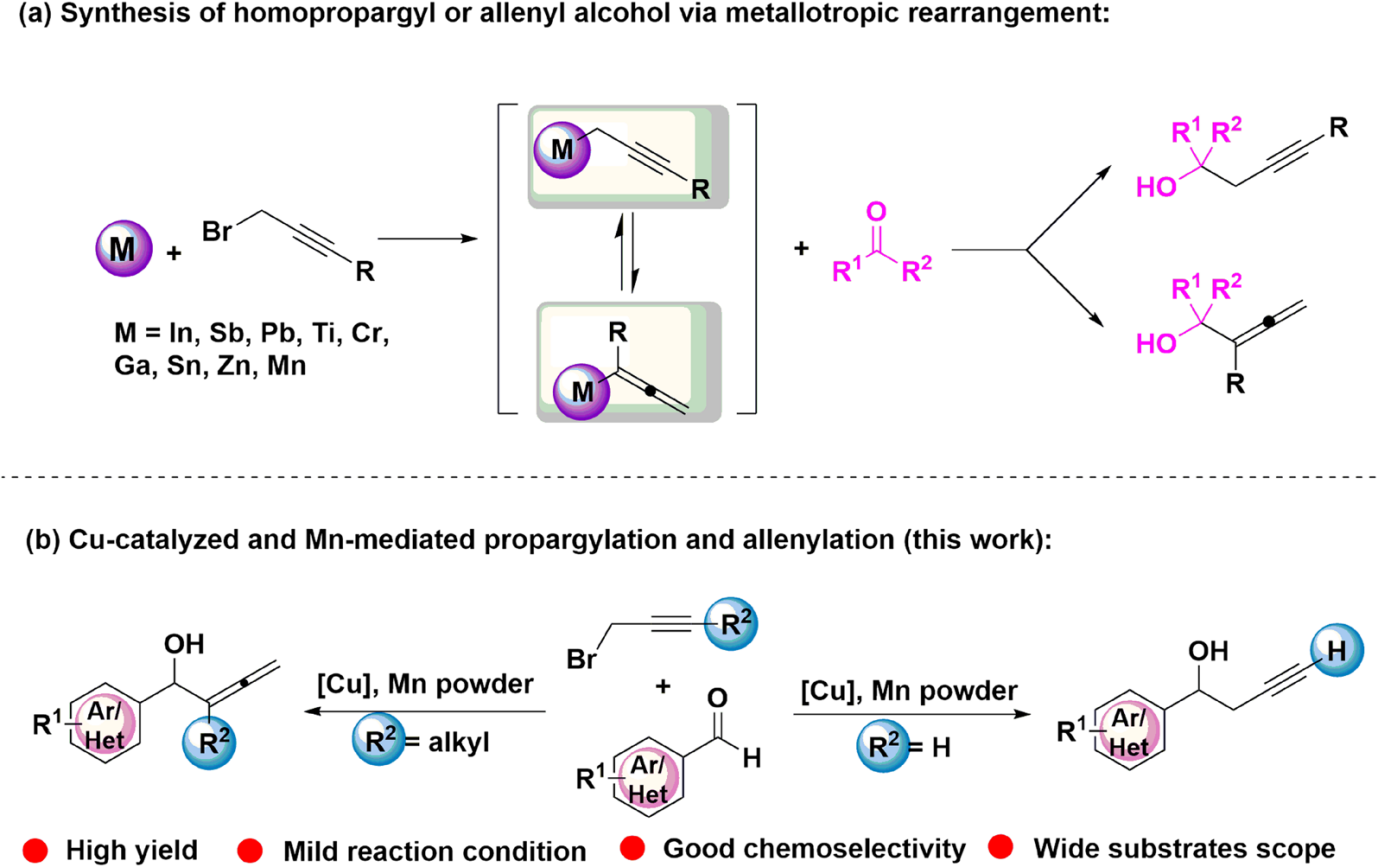
Previous studies and our concept

In this paper, we developed the first example of Cu-catalyzed and Mn-mediated propargylation and allenylation of aldehydes with propargyl bromides under a novel catalytic system, which is covered with advantages of high efficiency, good chemo-selectivity, and wide substrates scopes under mild reaction conditions (Fig. 1).

We initiated our investigation using benzaldehyde (**1a**) and propargyl bromide (**2a**) as model substrates which catalyzed by copper salts and Mn powder (Table 1). Without Mn, only trace amount of desired product was observed which indicated that Mn powder is indispensable (Table 1, entry 1). While in the absence of CuBr_2_, 16% of **3a** was produced which demonstrated the great importance of Cu catalyst (entry 2). Screening of different solvents illustrated that MeCN is the best reaction medium, giving the desired product **3a** in 47% yield (entry 3). While, only trace amount of product was observed in THF or DCM and 24% in EtOH (entries 4–6). The yield of products dropped sharply when the reaction was carried out in the open system (entry 7). Meanwhile, without the addition of CF_3_COOH, only 13% yield of **3a** was achieved (entry 8). Subsequently, extensive experiments were conducted to investigate the effects of different copper salts on the reaction. Series of Cu catalysts, including CuSO_4_, CuCl, CuCl_2_, CuBr and CuI were tested and CuCl gave the best result (entries 9–13). Adding 5 equiv. Mn powder, a remarkable increase has been presented (entry 14). Simultaneously, a light increase of yield was observed by increasing the amount of catalyst (entry 15). Further studies indicated that extending the reaction time to 24 h, **1a** can be transformed to **3a** completely under the standard conditions (entry 16).

**Table 1:** The effect of different parameters on the reaction of **1a** and **2a**.^a^


Entry	[Cu]	Solvent	Mn	Time/h	Yield/% of **3a**^b^
1	CuBr_2_	MeCN	–	12	Trace
2	–	MeCN	Mn	12	16
3	CuBr_2_	MeCN	Mn	12	47
4	CuBr_2_	THF	Mn	12	Trace
5	CuBr_2_	DCM	Mn	12	Trace
6	CuBr_2_	EtOH	Mn	12	24
7^*c*^	CuBr_2_	MeCN	Mn	12	24
8^*d*^	CuBr_2_	MeCN	Mn	12	13
9	CuSO_4_	MeCN	Mn	12	59
10	CuCl	MeCN	Mn	12	83
11	CuCl_2_	MeCN	Mn	12	63
12	CuBr	MeCN	Mn	12	74
13	CuI	MeCN	Mn	12	41
14^e^	CuCl	MeCN	Mn	12	75
15^f^	CuCl	MeCN	Mn	12	33
16	CuCl	MeCN	Mn	24	> 99

^a^Reaction conditions: All reactions were performed with **1a** (0.5 mmol), **2a** (1.5 equiv.), copper catalyst (10 mol%), Mn powder (3 equiv.), CF_3_COOH (25 mol%), solvent (2 mL), at room temperature under N_2_ atmosphere. ^b^ Yield was determined by GC with dodecane as internal standard based on **1a**. ^c^ Reaction in the air. ^d^ Without CF_3_COOH. ^e^ 5.0 equiv. of Mn was added. ^f^ CuCl (20 mol%) was added.

**Table 2:**
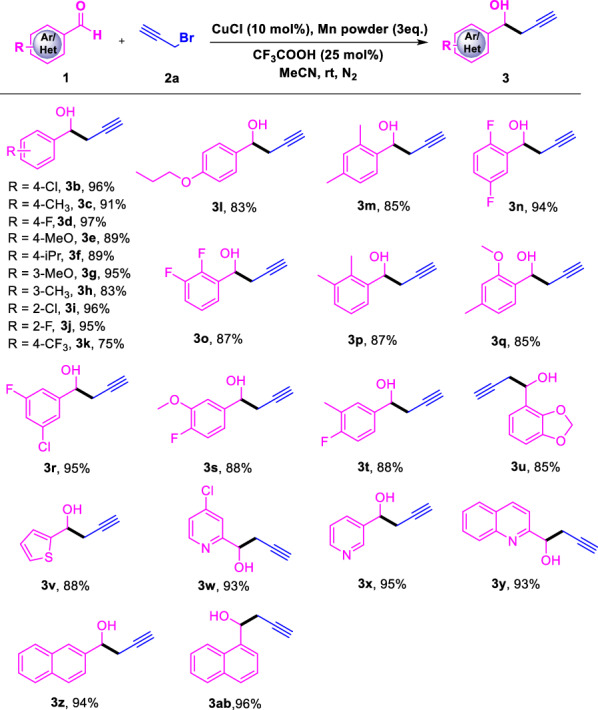
Cu-catalyzed and Mn-mediated propargylation of different aldehydes^a^

^a^ Standard condition: a solution of **1** (0.5 mmol), **2a** (1.5 equiv.), CuCl (10 mol%), Mn powder (3 equiv.) and CF_3_COOH (0.25 equiv.) in MeCN (2.0 mL) was reacted conducted at room temperature under N_2_ atmosphere for 24 h

**Table 3:**
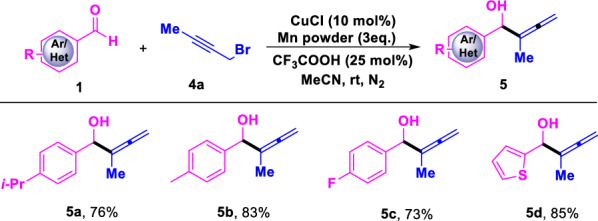
Cu-catalyzed and Mn-mediated allenylation of different aldehydes^a^

^a^ Standard condition: a solution of **1** (0.5 mmol), **4a** (1.5 equiv.), CuCl (10 mol%), Mn powder (3 equiv.) and CF_3_COOH (0.25 equiv.) in MeCN (2.0 mL) was reacted conducted at room temperature under N_2_ atmosphere for 24 h

With the optimized setup in hand, we next explored the substrates scope of aldehydes with different functional groups as shown in Table 2. It is pleasing that substrates bearing both electron-donating groups (EDGs) and electron-withdrawing groups (EWGs) can proceed smoothly. For example, substrates **3c**, **3e**, **3f**, **3g**, **3h**, **3i**, **3k** and **3o** with alkyl and alkoxy groups can be transformed to the corresponding products in excellent yield. Substrates containing the halogen (**3b**, **3d**, **3i**, **3j**) can also deliver the corresponding products with excellent yields. In addition, disubstituted benzaldehydes, such as 2,4-dimethyl (**3m**), 2,3-dimethyl (**3p**), 2,5-difluoro (**3n**), 2,3-difluoro (**3o**), 2-methoxy-4-methyl (**3q**) 3-chloro-5-fluoro (**3r**), 3-methoxy-4-fluoro (**3s**) and 3-methyl-4-fluor (**3t**) benzaldehydes were found to be compatible with the reaction in 85%- 95% yields. To further expand the scopes of the present catalytic system, reactions of heteroaromatic aldehydes including thiophenecarboxaldehyde (**3v**), pyridylaldehydes (**3w** and **3x**) and quinolinecarboxaldehyde (**3y**) which contain aromatic heterocycle in the molecules were also explored. Interesting, all of these substrates were compatible with the reaction conditions and produced the homopropargyl alcohols in excellent yield. Naphthyl compounds is also effective for the transformation converted to **3z** and **3ab** in the yield of 94% and 96% respectively.

When 1-bromo-2-butyne (**4a**) was used instead of propargyl bromide, the rearrangement product allenyl alcohol was achieved with good yield under the same reaction conditions (Table 3). Importantly, the direct propargylation product was not detected in this catalytic system, which indicated that the chemo-selectivity for this reaction is quite good. For example, substrates (**5a–5c**) which substituted by isopropyl-, methyl- and fluoro- groups on the aromatic ring, reacted well and provided the corresponding products in moderate yields. In addition, heteroaromatic aldehyde (**5d**) is also worked for the transformation and an allenyl substituted alcohol (**5e**) was obtained with 85% yield.

**Fig. 2:**
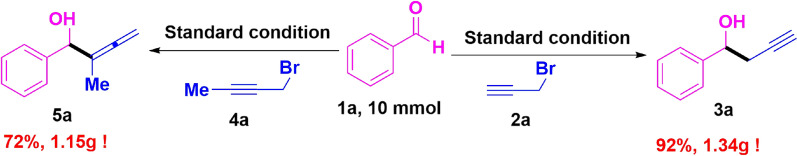
Gram-scale experiment

To demonstrate the synthetic applications of our protocols, we tried to scale up the reaction of benzaldehyde (**1a**) with 3-bromo-1-propyne (**2a**) or 1-bromo-2-pentyne (**4a**) independently under standard conditions (Fig. 2). The corresponding products **3a** or **5a** was obtained in a gram-scale, which highlightened the potential applicability of this transformation in organic synthesis.

**Fig. 3:**
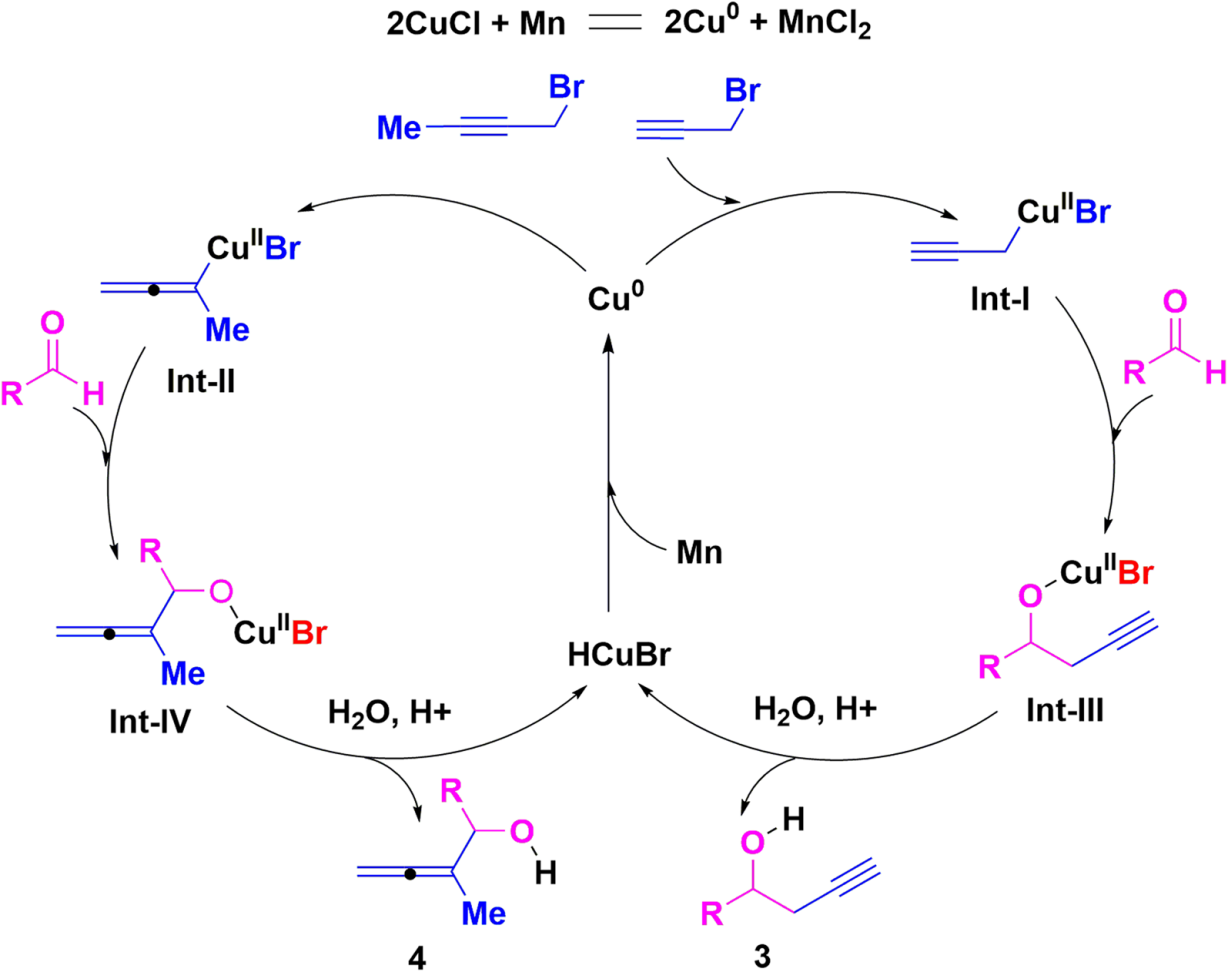
Proposed mechanism

Based on the above results and studies reported in the previous reference, a tentative mechanism for the Cu-Catalyzed, Mn-mediated propargylation and allenylation of aldehydes with propargyl bromides was proposed in Fig. 3 [52–56]. Mn, which is severed as a strong reducing agent, reduced the Cu^I^ to Cu^0^ in an active form in situ. Insertion of Cu^0^ to propargyl bromides gives the crucial intermediate progargyl metal species (**Int-I**) and allenyl metal species (**Int-II**). Then, nucleophilic addition of aldehydes conducted smoothly with metal species to deliver the **Int-III** and **Int-IV**. Finally, desired products were obtained in the presence of CF_3_COOH. The Cu^II^ complex was reduced to Cu^0^ with Mn powder to continue the next catalytic cycle.

In conclusion, the practical propargylation and allenylation of propargyl bromide has been discovered. The unique combination of the Cu catalyst and Mn powder present a novel and effective catalyst system in the preparation of homopropargylation alcohols and allenyl alcohols. Wide substrates compatibility has been exhibited with a variety of different substituent. This process represents a rare example of propargylation reaction and opens a new area of research. Further mechanistic studies and synthetic applications of this reaction are under progress in our laboratory.

## Experiment

### Procedure for the synthesis of homopropargyl alcohol

In a 10 mL Schlenk tube, aldehyde (0.5 mmol) was added to a stirred solution of 3-bromo-1-propyne (1.5 eq.), CuCl (10 mol%), Mn powder (3.0 eq.), and CF_3_COOH (25 mol%) in MeCN (2 mL) at room temperature under N_2_ atmosphere. After 24 h, the mixture was extracted with EtOAc (3 × 10 mL). The combined EtOAc layer was distilled and the crude product was then purified via column chromatograph.

### Procedure for the synthesis of allenyl alchols

In a 10 mL Schlenk tube, aldehyde (0.5 mmol) was added to a stirred solution of 1-bromo-2-pentyne (1.5 eq.) (1.5 eq.), CuCl (10 mol%), Mn powder (3.0 eq.), and CF_3_COOH (25 mol%) in MeCN (2 mL) at room temperature under N_2_ atmosphere. After 24 h, the mixture was extracted with EtOAc (3 × 10 mL). The combined EtOAc layer was distilled and the crude product was then purified via column chromatograph.

#### 1-phenylbut-3-yn-1-ol (**3a**) [57]

98% yield (71.6 mg), colourless oil. ^1^H NMR (400 MHz, CDCl_3_) δ 7.46–7.34 (m, 4H), 7.30 (ddd, J = 8.5, 3.6, 1.6 Hz, 1H), 4.88 (t, J = 5.4 Hz, 1H), 2.71–2.56 (m, 2H), 2.45 (s, 1H), 2.19–1.96 (m, 1H); ^13^C NMR (100 MHz, CDCl_3_) δ 142.4, 128.5, 128.0, 125.8, 80.7, 72.3, 71.0, 29.5.

#### 1-(4-chlorophenyl)but-3-yn-1-ol (**3b**) [57]

96% yield (86.7 mg), colorless oil. ^1^H NMR (400 MHz, CDCl_3_) δ 7.33–7.25 (m, 4H), 4.80 (t, J = 5.1 Hz, 1H), 2.81 (s, 1H), 2.58 (dd, J = 6.4, 2.5 Hz, 2H), 2.06 (dd, J = 3.4, 1.7 Hz, 1H). ^13^C NMR (100 MHz, CDCl_3_) δ 140.9, 133.7, 128.6, 127.2, 80.3, 71.6, 71.4, 29.4.

#### 1-(p-tolyl)but-3-yn-1-ol (**3c**) [57]

91% yield (72.8 mg), colorless oil. ^1^H NMR (400 MHz, CDCl_3_) δ 7.25 (d, J = 7.7 Hz, 2H), 7.14 (d, J = 7.7 Hz, 2H), 4.79 (s, 1H), 2.58 (dd, J = 11.1, 8.7 Hz, 3H), 2.33 (s, 3H), 2.03 (s, 1H). ^13^C NMR (100 MHz, CDCl_3_) δ 139.6, 137.7, 129.2, 125.8, 80.9, 72.2, 70.9, 29.3, 21.2.

#### 1-(4-fluorophenyl)but-3-yn-1-ol (**3d**) [57]

97% yield (79.6 mg), colorless oil. ^1^H NMR (400 MHz, CDCl_3_) δ 7.46–7.32 (m, 2H), 7.05 (t, J = 8.7 Hz, 2H), 4.86 (t, J = 5.5 Hz, 1H), 2.62 (dd, J = 6.3, 2.6 Hz, 2H), 2.49 (d, J = 2.5 Hz, 1H), 2.08 (t, J = 2.6 Hz, 1H). ^13^C NMR (100 MHz, CDCl_3_) δ 162.4 (d, J = 245 Hz), 138.2 (d, J = 3 Hz), 127.5 d, J = 8 Hz), 115.4 (d, J = 21 Hz), 80.4, 71.7, 71.2, 29.6.

#### 1-(4-methoxyphenyl)but-3-yn-1-ol (**3e**) [57]

89% yield (78.4 mg), colorless oil. ^1^H NMR (400 MHz, CDCl_3_) δ 7.29 (d, J = 8.6 Hz, 2H), 6.95–6.80 (m, 2H), 4.80 (t, J = 6.4 Hz, 1H), 3.79 (s, 3H), 2.64–2.58 (m, 2H), 2.05 (t, J = 2.6 Hz, 1H). ^13^C NMR (100 MHz, CDCl_3_) δ 159.3, 134.8, 127.1, 113.9, 80.9, 72.0, 70.9, 55.3, 29.3.

#### 1-(4-isopropylphenyl)but-3-yn-1-ol (**3f**) [57]

89% yield (83.7 mg), colorless oil. ^1^H NMR (400 MHz, CDCl_3_) δ 7.30 (d, J = 8.1 Hz, 2H), 7.21 (d, J = 8.2 Hz, 2H), 4.82 (s, 1H), 2.90 (dt, J = 13.8, 6.9 Hz, 1H), 2.62 (dd, J = 6.4, 2.6 Hz, 2H), 2.51 (s, 1H), 2.06 (t, J = 2.6 Hz, 1H), 1.24 (d, J = 6.9 Hz, 6H). ^13^C NMR (100 MHz, CDCl_3_) δ 148.7, 139.9, 126.6, 125.8, 81.0, 72.3, 70.9, 33.9, 29.3, 24.0.

#### 1-(3-methoxyphenyl)but-3-yn-1-ol (**3g**) [57]

95% yield (83.6 mg), colorless oil. ^1^H NMR (400 MHz, CDCl_3_) δ 7.27 (dd, J = 10.3, 5.9 Hz, 1H), 6.99–6.93 (m, 2H), 6.84 (ddd, J = 8.2, 2.5, 1.0 Hz, 1H), 4.85 (t, J = 6.3 Hz, 1H), 3.81 (s, 3H), 2.69–2.59 (m, 2H), 2.51 (s, 1H), 2.08 (t, J = 2.6 Hz, 1H). ^13^C NMR (100 MHz, CDCl_3_) δ 159.7, 144.2, 129.6, 118.1, 113.5, 111.3, 80.7, 72.3, 71.0, 55.3, 29.4.

#### 1-(m-tolyl)but-3-yn-1-ol (**3h**) [57]

83% yield (66.5 mg), colorless oil. ^1^H NMR (400 MHz, CDCl_3_) δ 7.24 (t, J = 7.5 Hz, 1H), 7.21–7.14 (m, 2H), 7.10 (d, J = 7.4 Hz, 1H), 4.82 (t, J = 6.4 Hz, 1H), 2.62 (dd, J = 6.4, 2.6 Hz, 2H), 2.51 (s, 1H), 2.35 (s, 3H), 2.06 (t, J = 2.6 Hz, 1H). ^13^C NMR (100 MHz, CDCl_3_) δ 142.5, 138.2, 128.8, 128.4, 126.4, 122.9, 80.9, 72.4, 70.9, 29.4, 21.5.

#### 1-(2-chlorophenyl)but-3-yn-1-ol (**3i**) [57]

96% yield (86.4 mg), colorless oil. ^1^H NMR (400 MHz, CDCl_3_) δ 7.62 (dd, J = 7.7, 1.4 Hz, 1H), 7.36–7.26 (m, 2H), 7.26–7.20 (m, 1H), 5.28 (dd, J = 7.8, 4.0 Hz, 1H), 2.80 (ddd, J = 16.9, 3.9, 2.7 Hz, 1H), 2.69 (s, 1H), 2.54 (ddd, J = 16.9, 7.8, 2.6 Hz, 1H), 2.10 (t, J = 2.6 Hz, 1H). ^13^C NMR (100 MHz, CDCl_3_) δ 139.7, 131.7, 129.4, 129.0, 127.1, 127.1, 80.3, 71.2, 68.7, 27.7.

#### 1-(2-fluorophenyl)but-3-yn-1-ol (**3j**) [57]

95% yield (79.9 mg), colorless oil. ^1^H NMR (400 MHz, CDCl_3_) δ 7.52 (td, J = 7.5, 1.5 Hz, 1H), 7.26 (ddd, J = 7.1, 4.6, 1.9 Hz, 1H), 7.16 (td, J = 7.5, 0.8 Hz, 1H), 7.02 (ddd, J = 10.4, 8.2, 0.9 Hz, 1H), 5.18 (dd, J = 7.2, 4.9 Hz, 1H), 2.74 (ddd, J = 16.8, 4.7, 2.6 Hz, 1H), 2.62 (ddd, J = 16.8, 7.6, 2.6 Hz, 2H), 2.07 (t, J = 2.6 Hz, 1H). ^13^C NMR (100 MHz, CDCl_3_) δ 160.0 (d, J = 244 Hz), 129.5, 129.3 (d, J = 8 Hz), 127.2 (d, J = 4 Hz), 124.3 (d, J = 3 Hz), 115.3 (d, J = 22 Hz), 80.3, 71.1, 66.4 (d, J = 2 Hz), 28.2.

#### 1-(4-(trifluoromethyl)phenyl)but-3-yn-1-ol (**3ak**) [57]

75% yield (80.0 mg), colorless oil. ^1^H NMR (400 MHz, CDCl_3_) δ 7.60 (d, J = 8.2 Hz, 2H), 7.49 (d, J = 8.1 Hz, 2H), 4.90 (t, J = 6.3 Hz, 1H), 2.83 (s, 1H), 2.65 – 2.59 (m, 2H), 2.08 (s, 1H). ^13^C NMR (100 MHz, CDCl_3_) δ 146.3, 130.0 (q, J = 32 Hz), 126.1, 125.4 (q, J = 4 Hz), 123.9 (q, J = 270 Hz), 79.9, 71.6 (d, J = 7 Hz), 29.4.

#### 1-(4-propoxyphenyl)but-3-yn-1-ol (**3l**) [57]

85% yield (86.8 mg), colorless oil. ^1^H NMR (400 MHz, CDCl_3_) δ 7.30 (d, J = 8.5 Hz, 2H), 6.88 (d, J = 8.5 Hz, 2H), 4.83 (t, J = 6.2 Hz, 1H), 3.91 (t, J = 6.6 Hz, 2H), 2.68–2.58 (m, 2H), 2.36 (s, 1H), 2.07 (d, J = 2.3 Hz, 1H), 1.80 (dd, J = 14.1, 7.0 Hz, 2H), 1.03 (t, J = 7.4 Hz, 3H). ^13^C NMR (100 MHz, CDCl_3_) δ 158.9, 134.4, 127.0, 114.4, 80.9, 72.1, 70.8, 69.5, 29.4, 22.6, 10.5.

#### 1-(2,4-dimethylphenyl)but-3-yn-1-ol (**3m**) [57]

85% yield (74.0 mg), colorless oil. ^1^H NMR (400 MHz, CDCl_3_) δ 7.36 (d, J = 7.9 Hz, 1H), 7.03 (d, J = 7.7 Hz, 1H), 6.95 (s, 1H), 5.04 (t, J = 6.4 Hz, 1H), 2.62–2.54 (m, 2H), 2.45 (d, J = 4.8 Hz, 1H), 2.30 (s, 3H), 2.29 (s, 3H), 2.05 (t, J = 2.6 Hz, 1H). ^13^C NMR (100 MHz, CDCl_3_) δ 137.6, 137.4, 134.6, 131.3, 127.0, 125.1, 81.1, 70.7, 68.8, 28.3, 21.0, 19.0.

#### 1-(2,5-difluorophenyl)but-3-yn-1-ol (**3n**) [57]

94% yield (85.6 mg), colorless oil. ^1^H NMR (400 MHz, CDCl_3_) δ 7.26 (ddd, J = 8.8, 5.8, 3.0 Hz, 1H), 7.10–6.79 (m, 2H), 5.24–5.07 (m, 1H), 2.83–2.48 (m, 3H), 2.10 (t, J = 2.6 Hz, 1H). ^13^C NMR (100 MHz, CDCl_3_) δ 158.9 (dd, J = 241, 2 Hz), 155.3 (dd, J = 238, 3 Hz), 131.2 (dd, J = 16, 7 Hz), 116.3 (dd, J = 24, 8 Hz), 115.5 (dd, J = 24, 9 Hz), 113.9 (dd, J = 25, 4 Hz), 79.7, 71.6, 65.9, 28.2 (d, J = 1 Hz).

#### 1-(2,3-difluorophenyl)but-3-yn-1-ol (**3o**) [57]

87% yield (79.2 mg), colorless oil. ^1^H NMR (400 MHz, CDCl_3_) δ 7.33–7.23 (m, 1H), 7.18–7.01 (m, 2H), 5.19 (dd, J = 6.9, 5.2 Hz, 1H), 2.82 (s, 1H), 2.74 (ddd, J = 16.8, 4.8, 2.6 Hz, 1H), 2.63 (ddd, J = 16.8, 7.4, 2.5 Hz, 1H), 2.08 (t, J = 2.5 Hz, 1H). ^13^C NMR (100 MHz, CDCl_3_) δ 150.2 (dd, J = 246, 12 Hz), 147.6 (dd, J = 246, 13), 131.9 (d, J = 10 Hz), 124.2 (dd, J = 7, 5 Hz), 121.8 (t, J = 3 Hz), 116.5 (d, J = 2 Hz), 79.8, 71.4, 66. 0 (t, J = 2 Hz), 28.2.

#### 1-(2,3-dimethylphenyl)but-3-yn-1-ol (**3p**) [57]

87% yield (75.7 mg), colorless oil. ^1^H NMR (400 MHz, CDCl_3_) δ 7.36 (d, J = 7.4 Hz, 1H), 7.17–7.04 (m, 2H), 5.15 (dd, J = 7.6, 5.0 Hz, 1H), 2.61–2.51 (m, 2H), 2.28 (s, 3H), 2.22 (s, 3H), 2.07 (d, J = 2.4 Hz, 1H), 1.97 (s, 1H). ^13^C NMR (100 MHz, CDCl_3_) δ 140.4, 137.0, 133.2, 129.4, 125.8, 122.9, 81.2, 70.7, 69.3, 28.3, 20.7, 14.7.

#### 1-(2-methoxy-4-methylphenyl)but-3-yn-1-ol (**3q**) [57]

85% yield (80.8 mg), colorless oil. ^1^H NMR (400 MHz, CDCl_3_) δ 7.25 (d, J = 7.6 Hz, 1H), 6.77 (d, J = 7.6 Hz, 1H), 6.68 (s, 1H), 5.09- 4.96 (m, 1H), 3.83 (d, J = 6.7 Hz, 3H), 2.98 (s, 1H), 2.67 (dddd, J = 24.2, 10.1, 6.3, 2.6 Hz, 2H), 2.34 (s, 3H), 2.03 (t, J = 2.6 Hz, 1H). ^13^C NMR (100 MHz, CDCl_3_) δ 156.2, 138.9, 127.4, 126.8, 121.2, 111.4, 81.5, 70.4, 68.9, 55.2, 27.5, 21.6.

#### 1-(3-chloro-5-fluorophenyl)but-3-yn-1-ol (**3r**) [57]

95% yield (94.1 mg), colorless oil. ^1^H NMR (400 MHz, CDCl_3_) δ 7.19 (s, 1H), 7.07–6.99 (m, 2H), 4.84 (t, J = 4.6 Hz, 1H), 2.65–2.61 (m, 1H), 2.59 (dd, J = 6.5, 3.0 Hz, 1H), 2.11 (t, J = 2.6 Hz, 1H), 1.68 (s, 1H). ^13^C NMR (100 MHz, CDCl_3_) δ 163.7 (d, J = 248 Hz), 146.2 (d, J = 7 Hz), 135.1 (d, J = 10 Hz), 121.9 (d, J = 4 Hz), 115.6 (d, J = 25 Hz), 111.4 (d, J = 22 Hz), 79.6, 71.8, 71.2 (d, J = 2 Hz), 29.4.

#### 1-(4-fluoro-3-methoxyphenyl)but-3-yn-1-ol (**3s**) [57]

88% yield (85.4 mg), colorless oil. ^1^H NMR (400 MHz, CDCl_3_) δ 7.09–7.01 (m, 2H), 6.88 (ddd, J = 8.3, 4.3, 2.1 Hz, 1H), 4.83 (t, J = 6.3 Hz, 1H), 3.89 (d, J = 5.9 Hz, 3H), 2.62 (dd, J = 6.4, 2.6 Hz, 2H), 2.55 (s, 1H), 2.09 (t, J = 2.6 Hz, 1H). ^13^C NMR (100 MHz, CDCl_3_) δ 151.914.7 (d, J = 244 Hz), 147.6 (d, J = 11 Hz), 138.8 (d, J = 3 Hz), 118.1 (d, J = 7 Hz), 115.8 (d, J = 19 Hz), 110.9 (d, J = 2 Hz), 80.4, 71.9, 71.3, 56.2, 29.6.

#### 1-(4-fluoro-3-methylphenyl)but-3-yn-1-ol (**3t**) [57]

88% yield (78.4 mg), colorless oil. ^1^H NMR (400 MHz, CDCl_3_) δ 7.24–7.08 (m, 2H), 6.97 (t, J = 8.9 Hz, 1H), 4.81 (t, J = 6.3 Hz, 1H), 2.61 (dd, J = 6.3, 2.4 Hz, 2H), 2.45 (s, 1H), 2.27 (s, 3H), 2.08 (s, 1H). ^13^C NMR (100 MHz, CDCl_3_) δ 160.9 (d, J = 243 Hz), 137.87, 128.9 (d, J = 2 Hz), 125.0, 124.7(d, J = 8 Hz), 114.9 (d, J = 22 Hz), 80.6, 71.8, 71.1, 29.5, 14.7(d, J = 4 Hz).

#### 2-(benzo[d][1,3]dioxol-4-yl)but-3-yn-1-ol (**3u**) [57]

85% yield (80.8 mg), colorless oil. ^1^H NMR (400 MHz, CDCl_3_) δ 6.95–6.89 (m, 1H), 6.84 (t, J = 7.8 Hz, 1H), 6.78 (dd, J = 7.6, 1.0 Hz, 1H), 5.96 (dd, J = 9.2, 1.1 Hz, 2H), 4.98 (dd, J = 10.2, 6.3 Hz, 1H), 2.84–2.58 (m, 3H), 2.06 (t, J = 2.6 Hz, 1H). ^13^C NMR (100 MHz, CDCl_3_) δ 147.4, 144.1, 124.1, 121.8, 119.3, 108.2, 101.0, 80.5, 70.9, 68.3, 27.6.

#### 1-(thiophen-2-yl)but-3-yn-1-ol (**3v**) [57]

88% yield (66.9 mg), colorless oil. ^1^H NMR (400 MHz, CDCl_3_) δ 7.32–7.22 (m, 1H), 6.99 (ddd, J = 11.1, 6.1, 2.5 Hz, 2H), 5.11 (d, J = 3.7 Hz, 1H), 2.79–2.68 (m, 3H), 2.11 (dd, J = 5.2, 2.6 Hz, 1H). ^13^C NMR (100 MHz, CDCl_3_) δ 146.2, 126.7, 125.0, 124.2, 80.1, 71.5, 68.5, 29.5.

#### 1-(4-chloropyridin-2-yl)but-3-yn-1-ol (**3w**) [57]

93% yield (84.1 mg), colorless oil. ^1^H NMR (400 MHz, CDCl_3_) δ 8.46 (d, J = 5.3 Hz, 1H), 7.49 (d, J = 1.7 Hz, 1H), 7.26 (dd, J = 5.4, 2.0 Hz, 1H), 4.88 (t, J = 6.0 Hz, 1H), 2.78–2.65 (m, 2H), 2.06 (t, J = 2.6 Hz, 1H), 1.25 (s, 1H). ^13^C NMR (100 MHz, CDCl_3_) δ 162.0, 149.4, 144.9, 123.3, 121.2, 80.0, 71.3, 71.1, 28.2.

#### 1-(pyridin-3-yl)but-3-yn-1-ol (**3x**) [57]

95% yield (69.9 mg), colorless oil. ^1^H NMR (400 MHz, CDCl_3_) δ 8.54 (d, J = 2.0 Hz, 1H), 8.47 (dd, J = 4.8, 1.5 Hz, 1H), 7.79 (dt, J = 7.9, 1.8 Hz, 1H), 7.33–7.26 (m, 1H), 4.92 (t, J = 6.4 Hz, 1H), 2.70–2.65 (m, 2H), 2.08 (t, J = 2.6 Hz, 1H), 1.35–1.23 (m, 1H). ^13^C NMR (100 MHz, CDCl_3_) δ 148.9, 147.6, 138.3, 133.9, 123.5, 79.9, 71.5, 70.0, 29.3.

#### 1-(quinolin-2-yl)but-3-yn-1-ol (**3y**) [57]

93% yield (92.0 mg), colorless oil. ^1^H NMR (400 MHz, CDCl_3_) δ 8.19 (d, J = 8.5 Hz, 1H), 8.09 (d, J = 8.5 Hz, 1H), 7.85 (d, J = 8.1 Hz, 1H), 7.73 (dd, J = 8.4, 1.4 Hz, 1H), 7.59–7.49 (m, 2H), 5.07 (t, J = 5.9 Hz, 1H), 2.80 (ddd, J = 5.9, 2.5, 1.7 Hz, 2H), 2.02 (t, J = 2.7 Hz, 1H), 1.25 (s, 1H). ^13^C NMR (100 MHz, CDCl_3_) δ 160.0, 146.5, 137.0, 129.9, 128.9, 127.8, 127.7, 126.7, 118.5, 80.5, 71.0, 71.0, 28.3.

#### 1-(naphthalen-2-yl)but-3-yn-1-ol (**3z**) [57]

94% yield (92.5 mg), colorless oil. ^1^H NMR (400 MHz, CDCl_3_) δ 7.87–7.70 (m, 4H), 7.56–7.36 (m, 3H), 4.98 (t, J = 6.3 Hz, 1H), 2.75 (s, 1H), 2.69 (dd, J = 6.4, 2.6 Hz, 2H), 2.05 (t, J = 2.6 Hz, 1H). ^13^C NMR (100 MHz, CDCl_3_) δ 139.9, 133.2, 133.2, 128.4, 128.1, 127.8, 126.3, 126.1, 124.8, 123.8, 80.8, 72.5, 71.2, 29.4.

#### 1-(naphthalen-1-yl)but-3-yn-1-ol (**3ab**) [57]

96% yield (94.1 mg), colorless oil. ^1^H NMR (400 MHz, CDCl_3_) δ 8.04 (d, J = 8.2 Hz, 1H), 7.89–7.83 (m, 1H), 7.79 (d, J = 8.2 Hz, 1H), 7.69 (d, J = 7.2 Hz, 1H), 7.54–7.44 (m, 3H), 5.63 (dd, J = 8.2, 4.2 Hz, 1H), 2.87 (ddd, J = 17.0, 4.2, 2.7 Hz, 1H), 2.73 (ddd, J = 17.0, 8.2, 2.6 Hz, 2H), 2.12 (t, J = 2.6 Hz, 1H). ^13^C NMR (100 MHz, CDCl_3_) δ 137.8, 133.8, 130.2, 129.1, 128.5, 126.3, 125.7, 125.4, 123.0, 122.8, 81.0, 71.3, 69.3, 28.7.

#### 1-(4-isopropylphenyl)-2-methyl-3λ5-buta-2,3-dien-1-ol (**5a**) [57]

76% yield (76.8 mg), colorless oil. ^1^H NMR (400 MHz, CDCl_3_) δ 7.30 (d, J = 8.0 Hz, 2H), 7.21 (d, J = 8.0 Hz, 2H), 5.07 (s, 1H), 4.96–4.85 (m, 2H), 2.90 (dt, J = 13.8, 6.9 Hz, 1H), 2.18 (s, 1H), 1.58 (t, J = 3.0 Hz, 3H), 1.24 (d, J = 7.0 Hz, 6H).^13^C NMR (100 MHz, CDCl_3_) δ 204.6, 148.6, 139.2, 126.6, 126.5, 102.7, 77. 9, 74.5, 33.9, 24.0, 14.7.

#### 2-methyl-1-(p-tolyl)-3λ^5^-buta-2,3-dien-1-ol (**5b**) [57]

83% yield (54.9 mg), colorless oil. ^1^H NMR (400 MHz, CDCl_3_) δ 7.25 (d, J = 8.0 Hz, 2H), 7.15 (d, J = 7.9 Hz, 2H), 5.05 (s, 1H), 4.96–4.82 (m, 2H), 2.34 (s, 3H), 2.30 (s, 1H), 1.56 (t, J = 3.1 Hz, 3H). ^13^C NMR (100 MHz, CDCl_3_) δ 204.7, 138.9, 137.5, 129.1, 126. 6, 102.7, 77.8, 74.5, 21.2, 14.7.

#### 1-(4-fluorophenyl)-2-methyl-3λ^5^-buta-2,3-dien-1-ol (**5c**) [57]

73% yield (73 mg), colorless oil. ^1^H NMR (400 MHz, CDCl_3_) δ 7.34 (dd, J = 8.4, 5.6 Hz, 2H), 7.03 (t, J = 8.7 Hz, 2H), 5.08 (s, 1H), 4.93–4.86 (m, 2H), 2.39 (s, 1H), 1.55 (t, J = 3.1 Hz, 3H). ^13^C NMR (100 MHz, CDCl_3_) δ 162.3(d, J = 244 Hz), 137.5 (d, J = 3 Hz), 128.3 (d, J = 8 Hz), 115.2 (d, J = 21 Hz), 102.6, 78.1, 77.4, 74.0, 14.5.

#### 2-methyl-1-(thiophen-2-yl)-3λ^5^-buta-2,3-dien-1-ol (**5d**) [57]

76% yield (63.1 mg), colorless oil. ^1^H NMR (400 MHz, CDCl_3_) δ 7.29–7.25 (m, 1H), 7.02 (d, J = 3.1 Hz, 1H), 6.99–6.95 (m, 1H), 5.35 (s, 1H), 4.99–4.86 (m, 2H), 2.34 (d, J = 4.4 Hz, 1H), 1.69 (t, J = 3.0 Hz, 3H). ^13^C NMR (100 MHz, CDCl_3_) δ 204.4, 146.1, 126.6, 125.2, 125.0, 102.6, 78.6, 70.7, 14.7.

## Conclusions

In conclusion, we have established the first Cu-catalyzed, Mn-mediated propargylation and allenylation of aldehydes with propargyl bromides. The unique combination of the Cu catalyst and Mn powder present a novel and effective catalyst system in the preparation of homopropargylation alcohols and allenyl alcohols. The overall transformation is highly efficient with mild conditions, large substrate scope, and excellent chem-selectivity.

## Supplementary Information

**Additional file 1.**

## Data Availability

All data generated or analyzed during this study are included in this published and its Additional file 1.
