# Supplementary material for: Cu-catalyzed, Mn-mediated propargylation and allenylation of aldehydes with propargyl bromides
Source: BMC Chem. 2022 Mar 18;16(1):14. doi: 10.1186/s13065-022-00803-3 (PMC8933908; doi:10.1186/s13065-022-00803-3)

**Additional Information**

**Cu-Catalyzed and Manganese Mediated Propargylation and Allenylation of Aldehydes Propargyl Bromide**

**Table of Contents**

NMR spectra for all compounds……………………………………………………S2

**^1^H NMR spectra of 1-phenylbut-3-yn-1-ol (3a)**


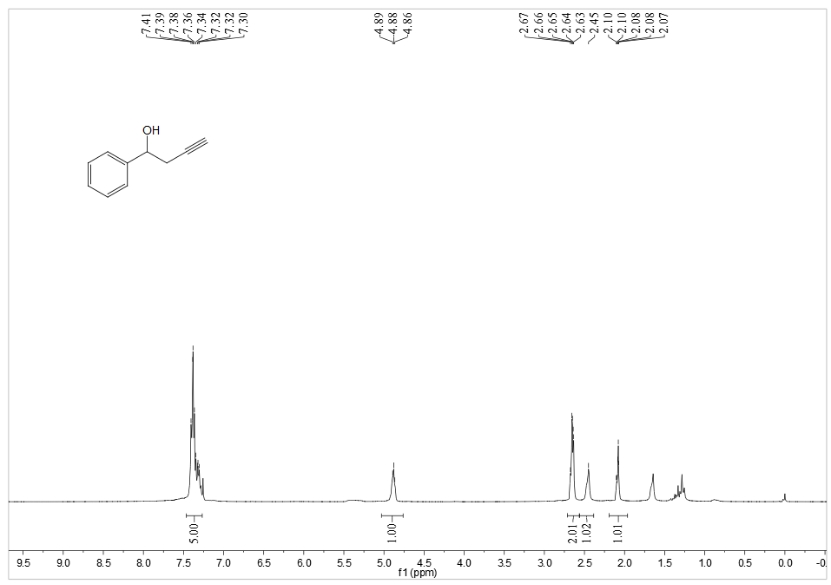


**^13^C NMR spectra of 1-phenylbut-3-yn-1-ol (3a)**


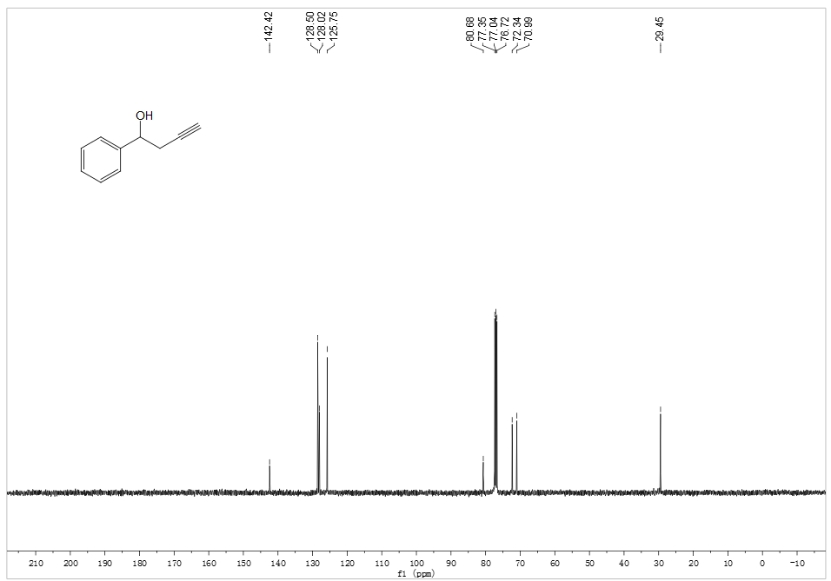


**^1^H NMR spectra of 1-(4-chlorophenyl)but-3-yn-1-ol (3b)**


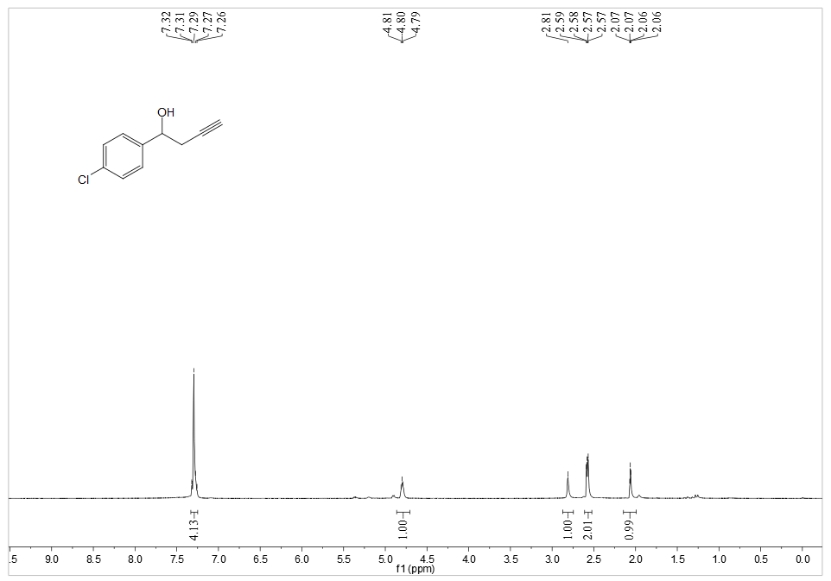


**^13^C NMR spectra of 1-(4-chlorophenyl)but-3-yn-1-ol (3b)**


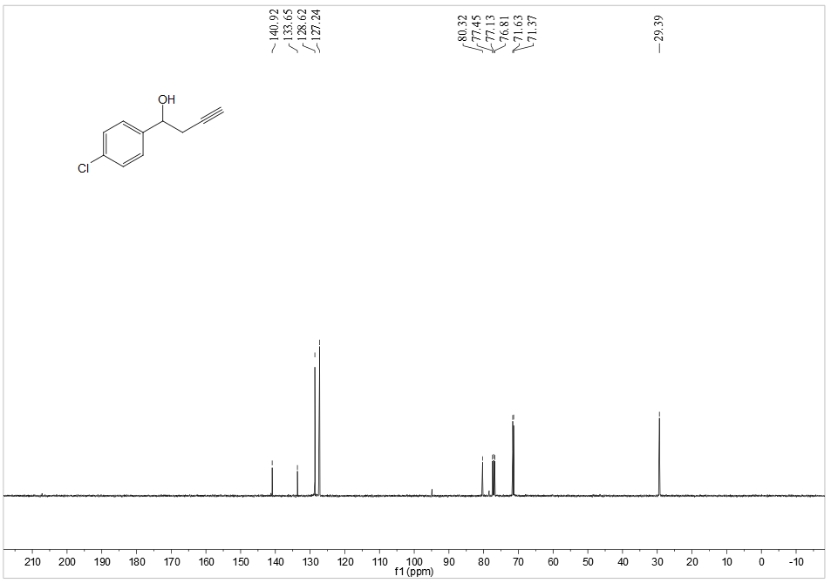


**^1^H NMR spectra of 1-(p-tolyl)but-3-yn-1-ol (3c)**


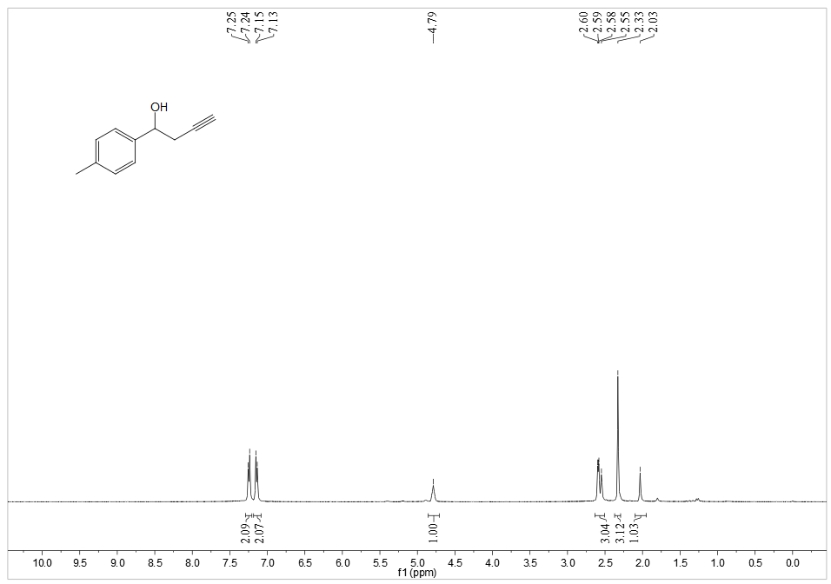


**^13^C NMR spectra of 1-(p-tolyl)but-3-yn-1-ol (3c)**


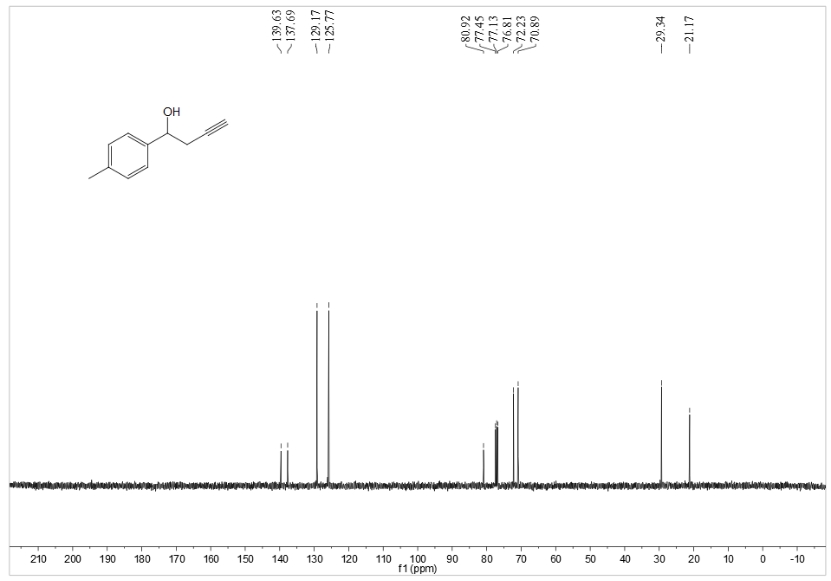


**^1^H NMR spectra of 1-(4-fluorophenyl)but-3-yn-1-ol (3d)**


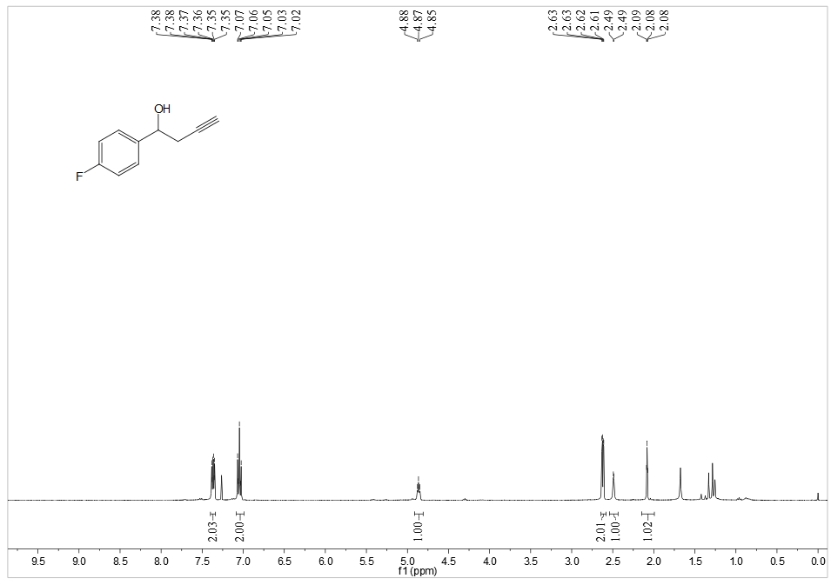


**^13^C NMR spectra of 1-(4-fluorophenyl)but-3-yn-1-ol (3d)**


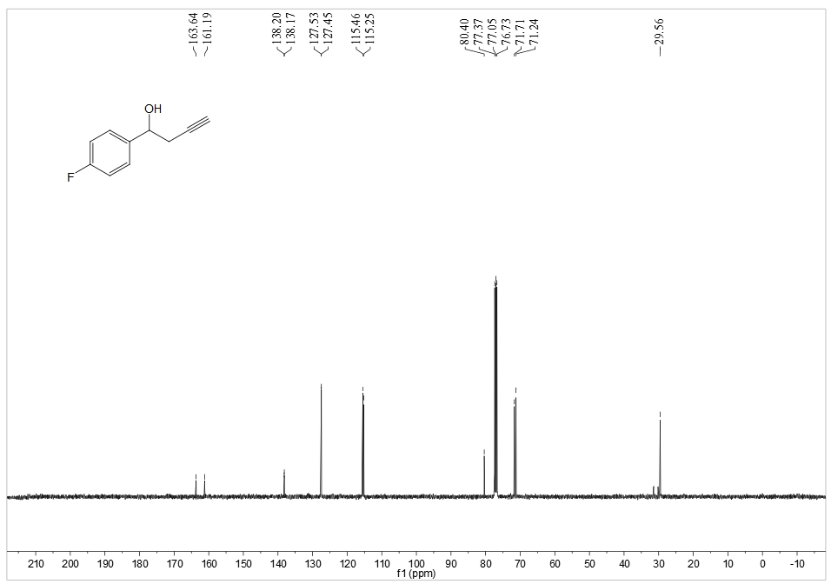


**^1^H NMR spectra of 1-(4-methoxyphenyl)but-3-yn-1-ol (3e)**


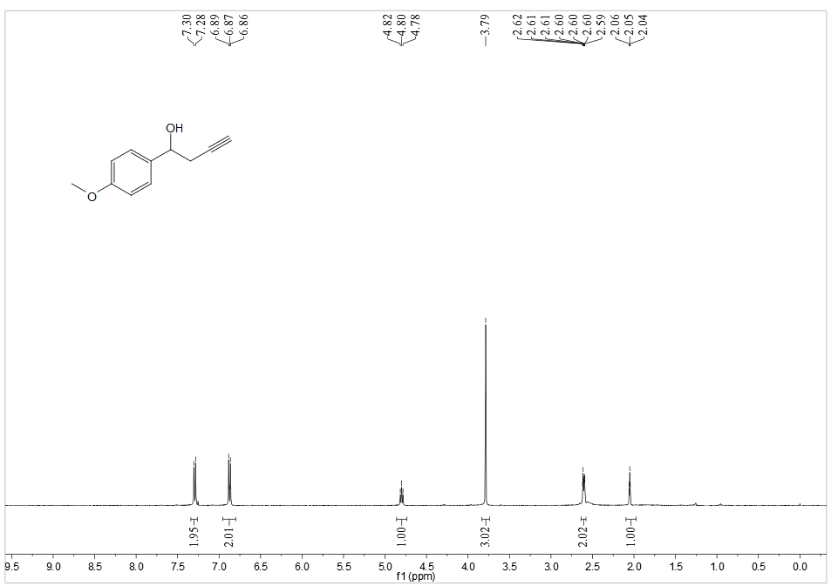


**^13^C NMR spectra of 1-(4-methoxyphenyl)but-3-yn-1-ol (3e)**


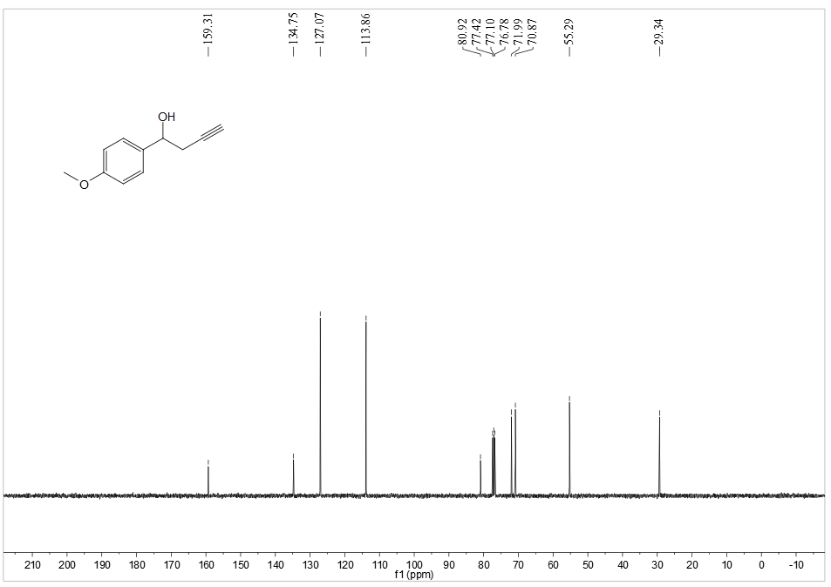


**^1^H NMR spectra of 1-(4-isopropylphenyl)but-3-yn-1-ol (3f)**


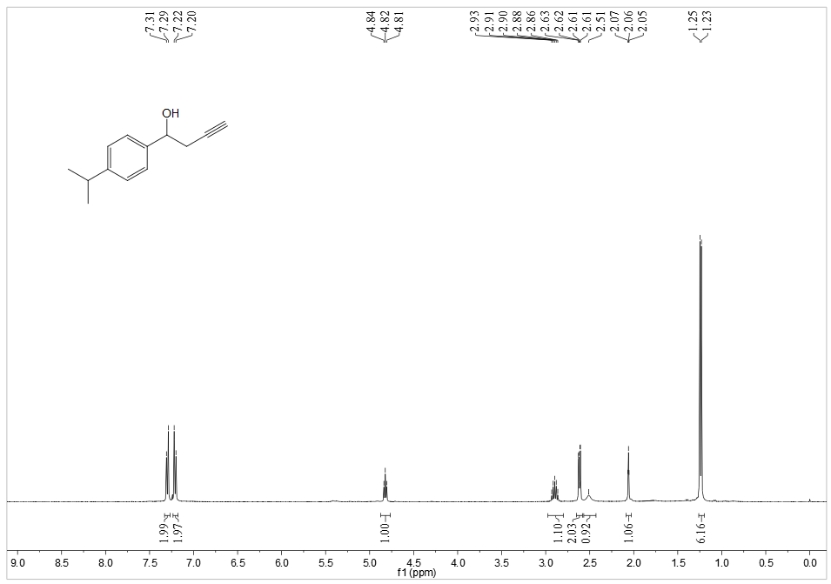


**^13^C NMR spectra of 1-(4-isopropylphenyl)but-3-yn-1-ol (3f)**


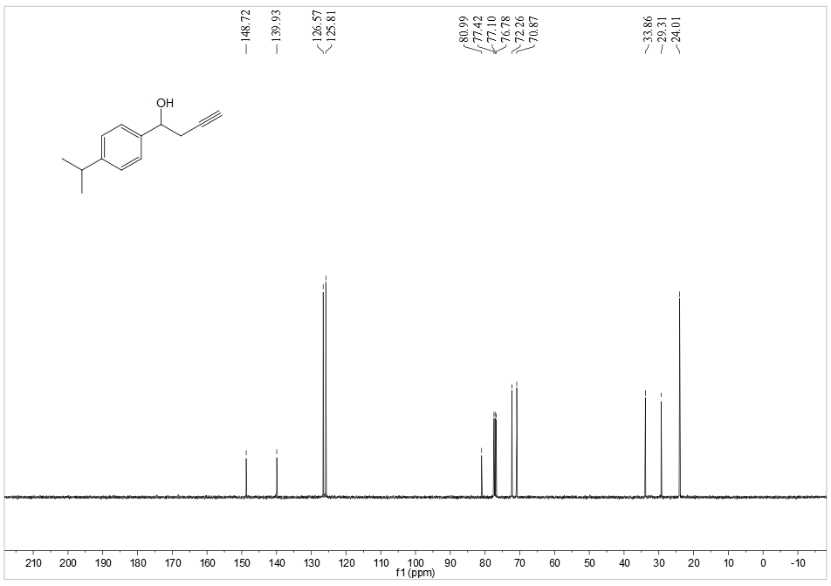


**^1^H NMR spectra of 1-(3-methoxyphenyl)but-3-yn-1-ol (3g)**


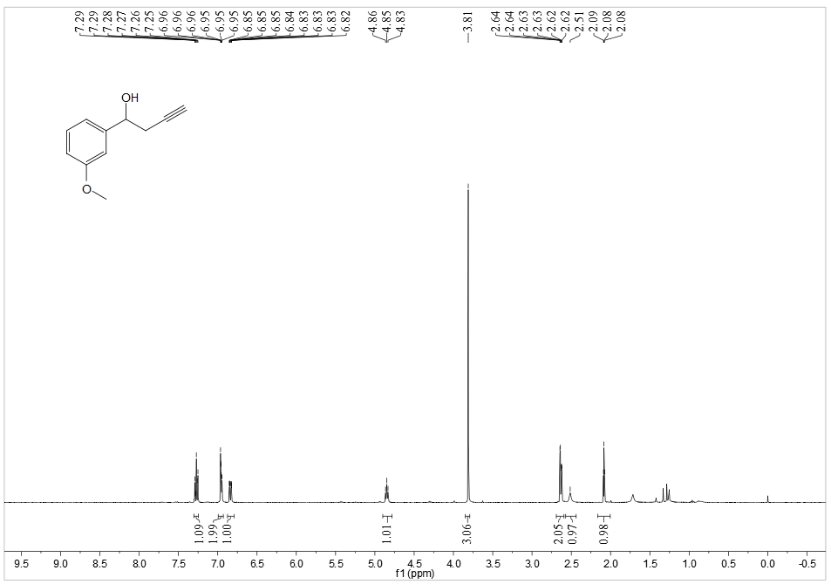


**^13^C NMR spectra of 1-(3-methoxyphenyl)but-3-yn-1-ol (3g)**


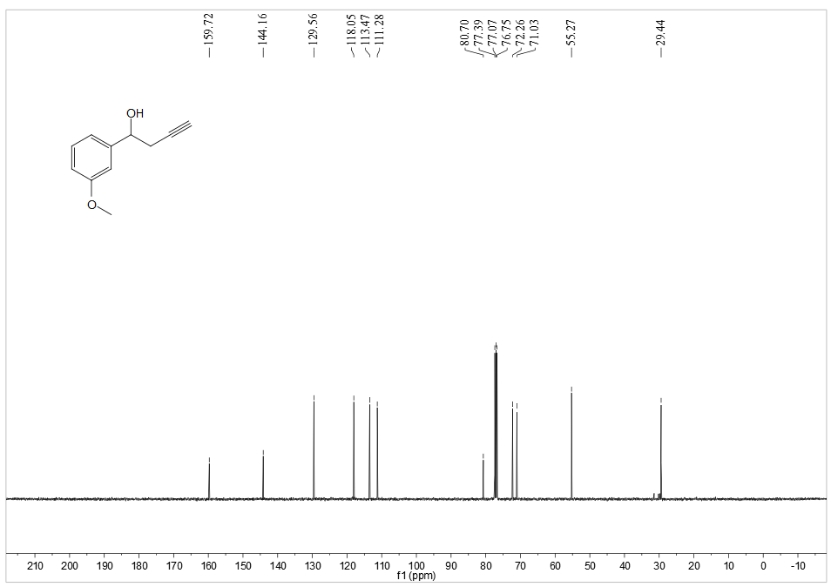


**^1^H NMR spectra of 1-(m-tolyl)but-3-yn-1-ol (3h)**


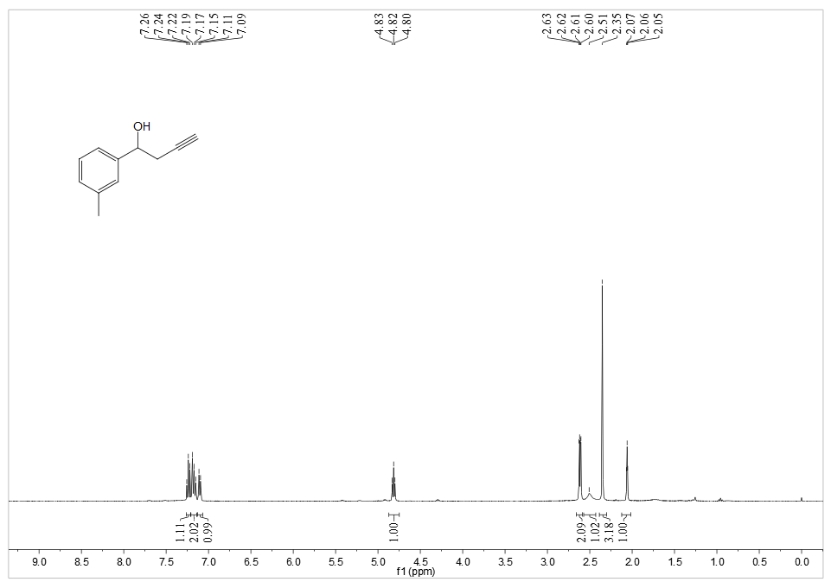


**^13^C NMR spectra of 1-(m-tolyl)but-3-yn-1-ol (3h)**


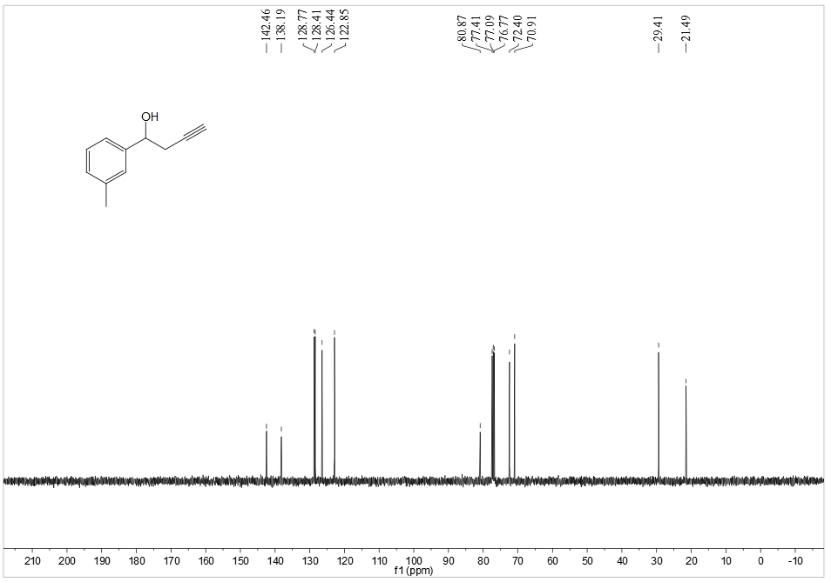


**^1^H NMR spectra of 1-(2-chlorophenyl)but-3-yn-1-ol (3i)**


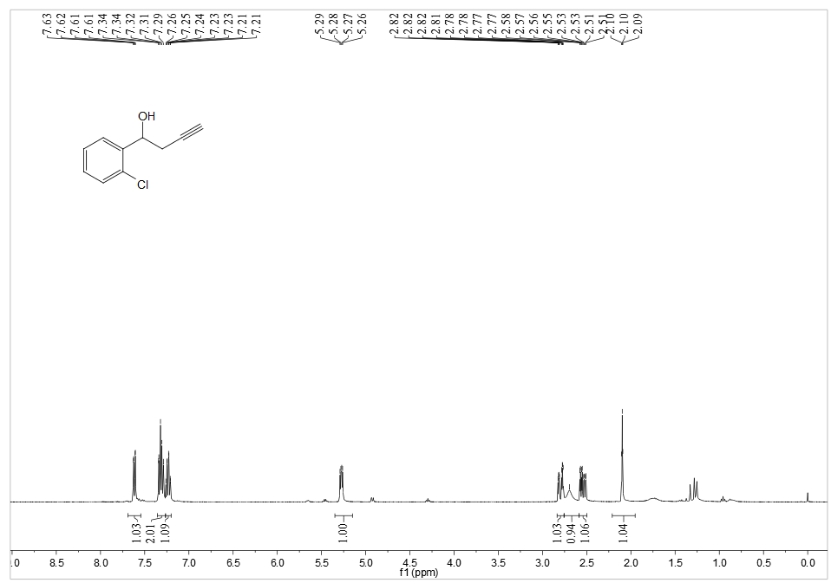


**^13^C NMR spectra of 1-(2-chlorophenyl)but-3-yn-1-ol (3i)**


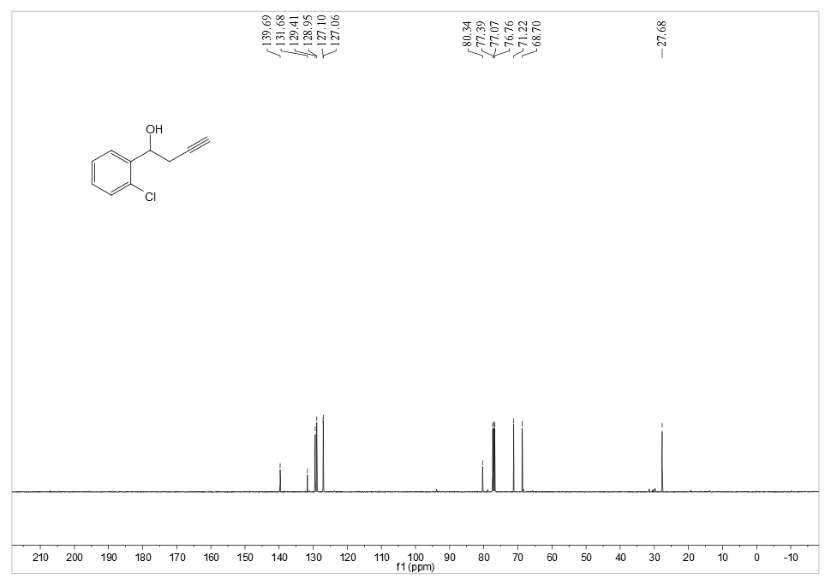


**^1^H NMR spectra of 1-(2-fluorophenyl)but-3-yn-1-ol (3j)**


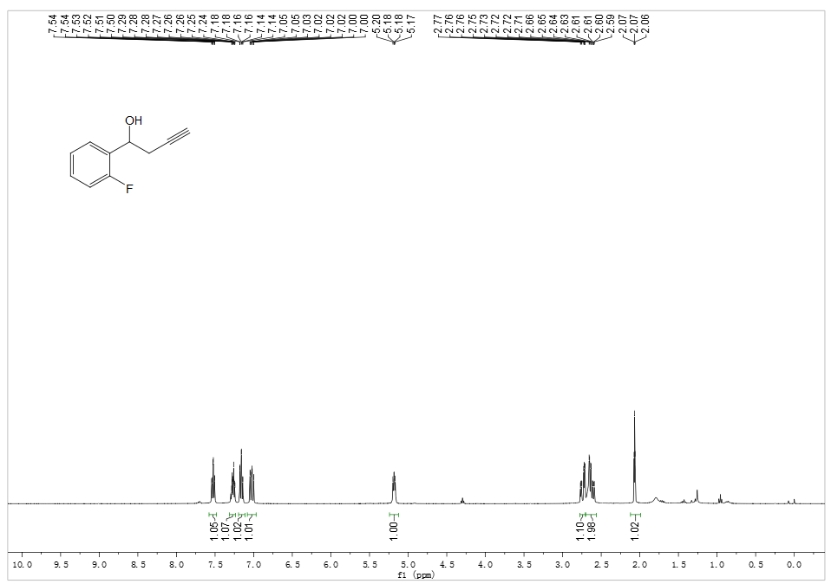


**^13^C NMR spectra of 1-(2-fluorophenyl)but-3-yn-1-ol (3j)**


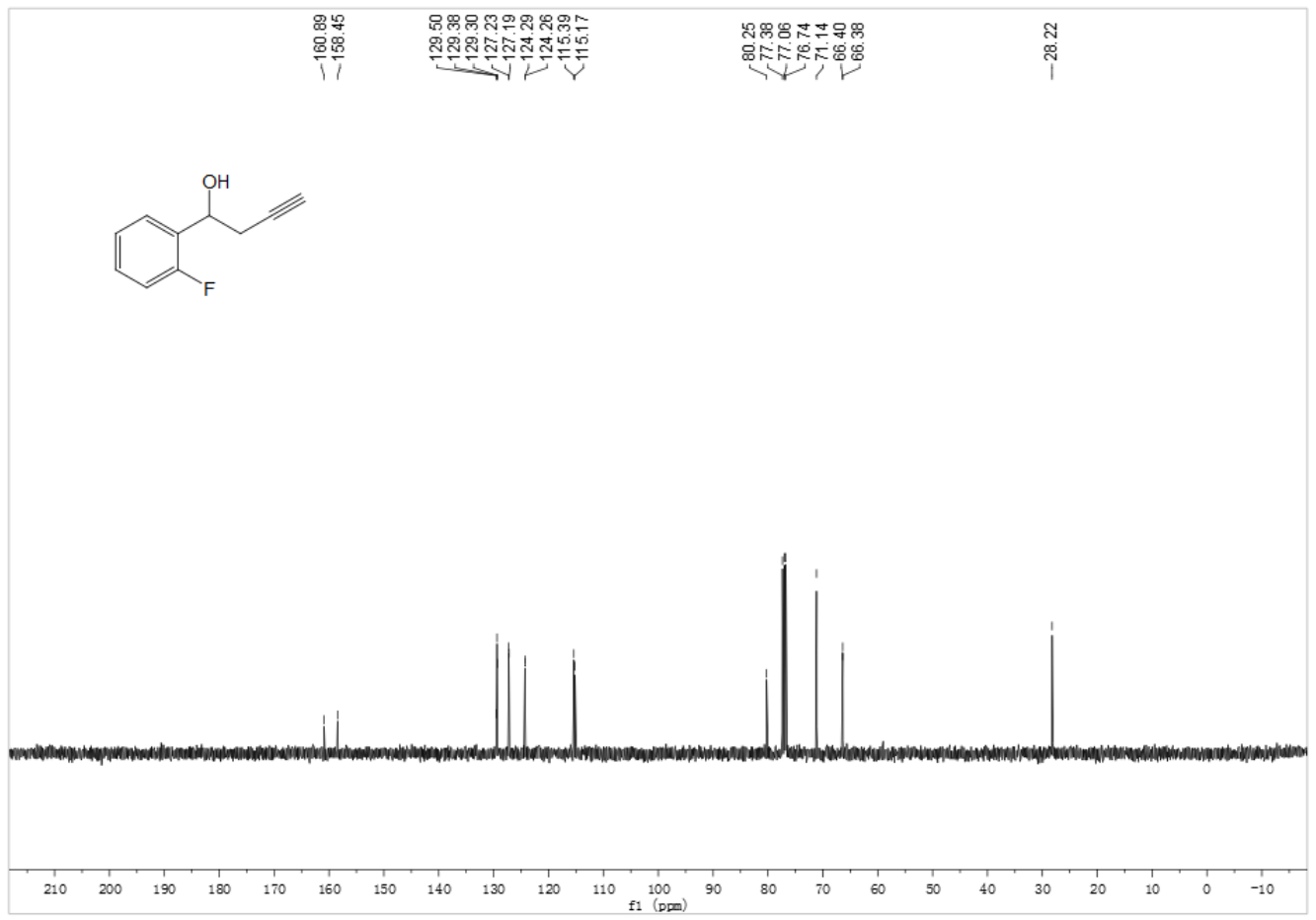


**^1^H NMR spectra of 1-(4-(trifluoromethyl)phenyl)but-3-yn-1-ol (3k)**


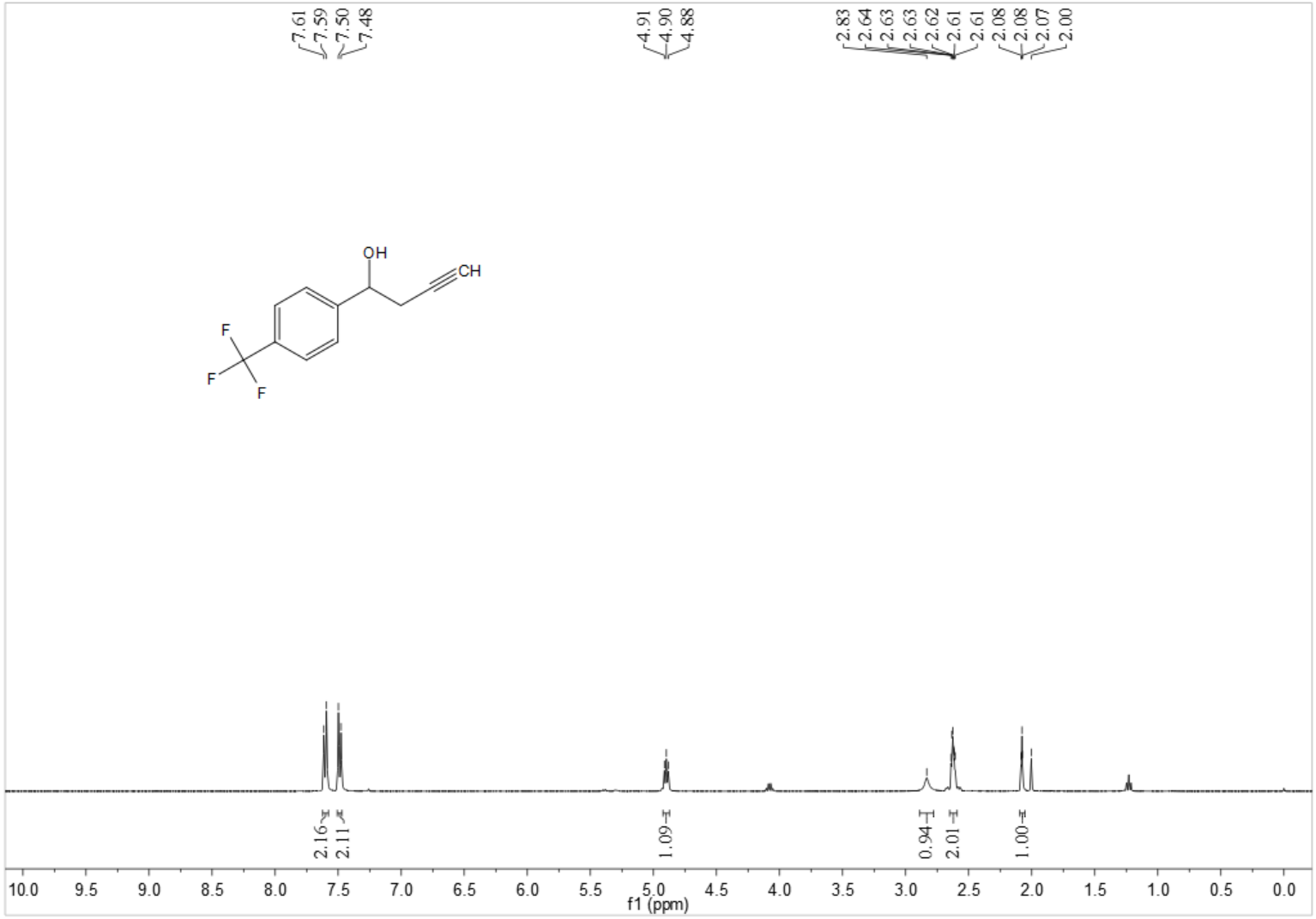


**^13^C NMR spectra of 1-(4-(trifluoromethyl)phenyl)but-3-yn-1-ol (3k)**


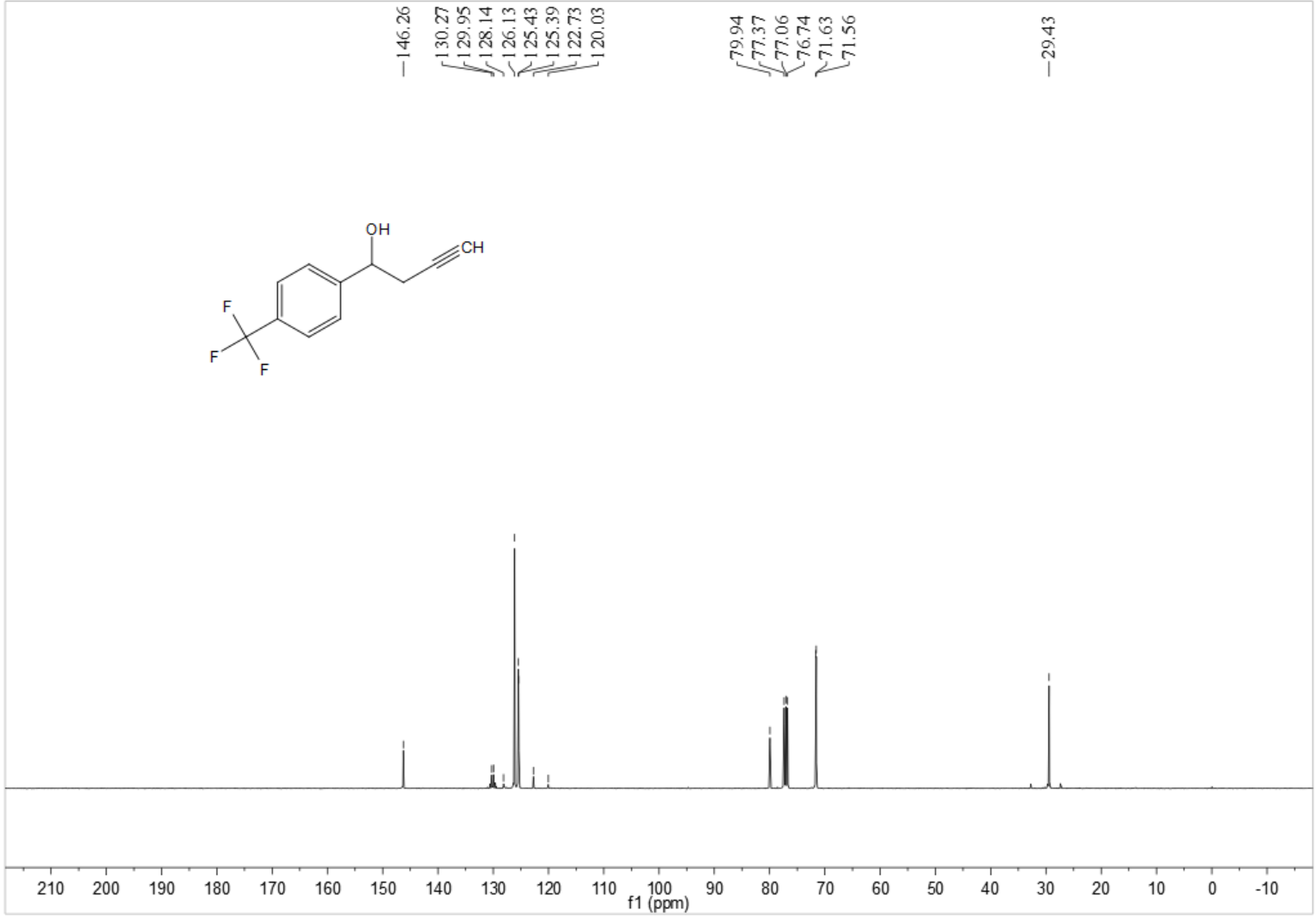


**^1^H NMR spectra of 1-(4-propoxyphenyl)but-3-yn-1-ol (3l)**


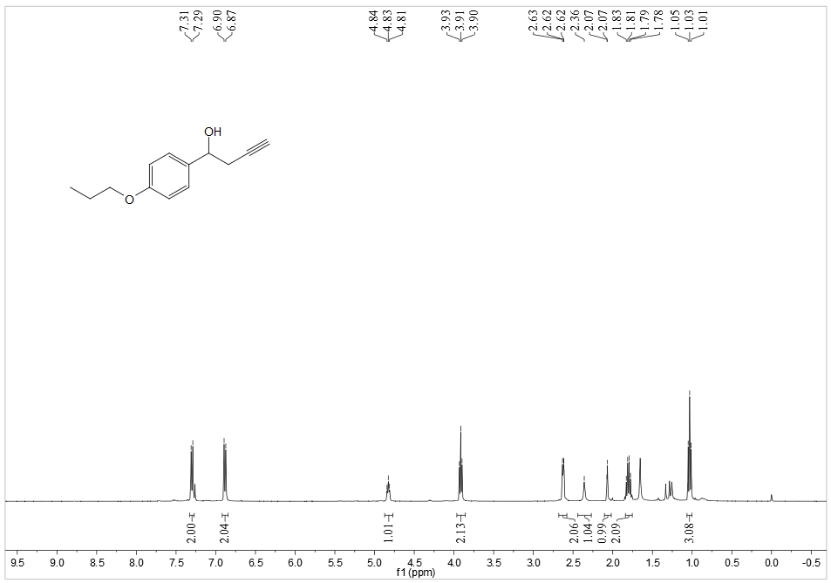


**^13^C NMR spectra of 1-(4-propoxyphenyl)but-3-yn-1-ol (3l)**


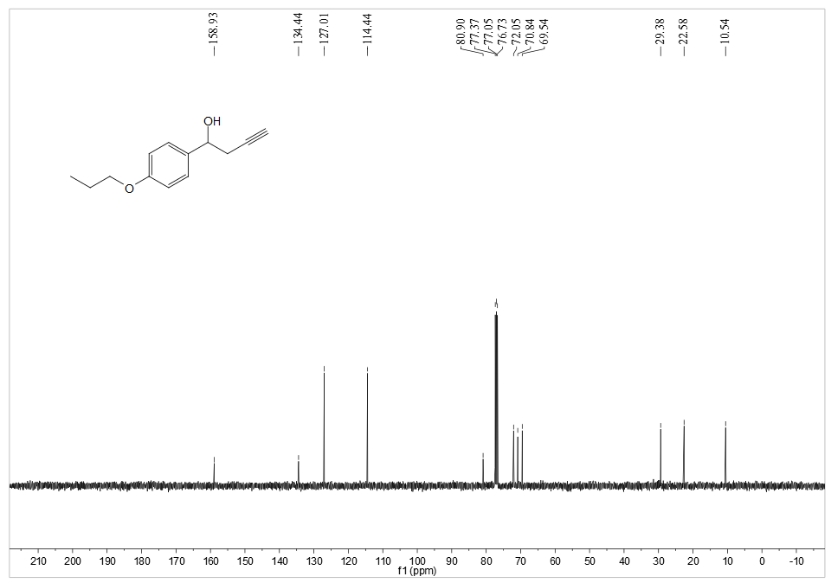


**^1^H NMR spectra of 1-(2,4-dimethylphenyl)but-3-yn-1-ol (3m)**
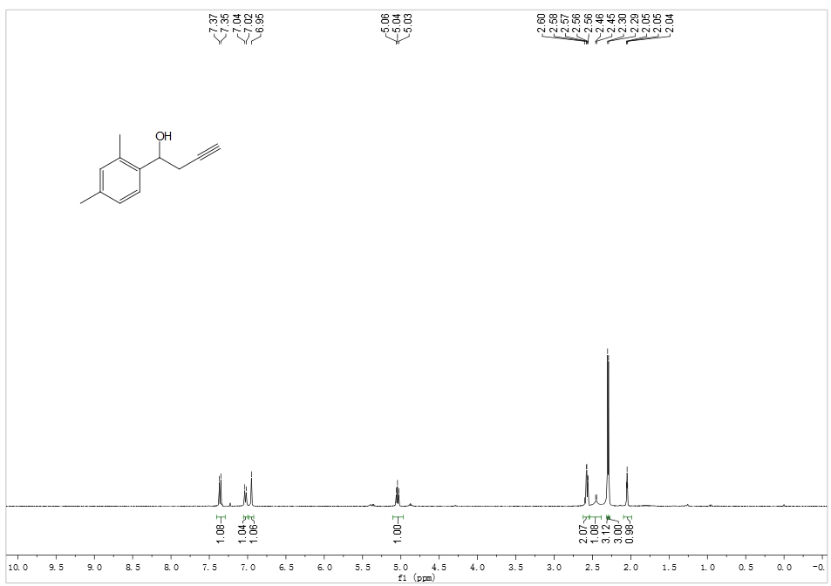


**^13^C NMR spectra of 1-(2,4-dimethylphenyl)but-3-yn-1-ol (3m)**
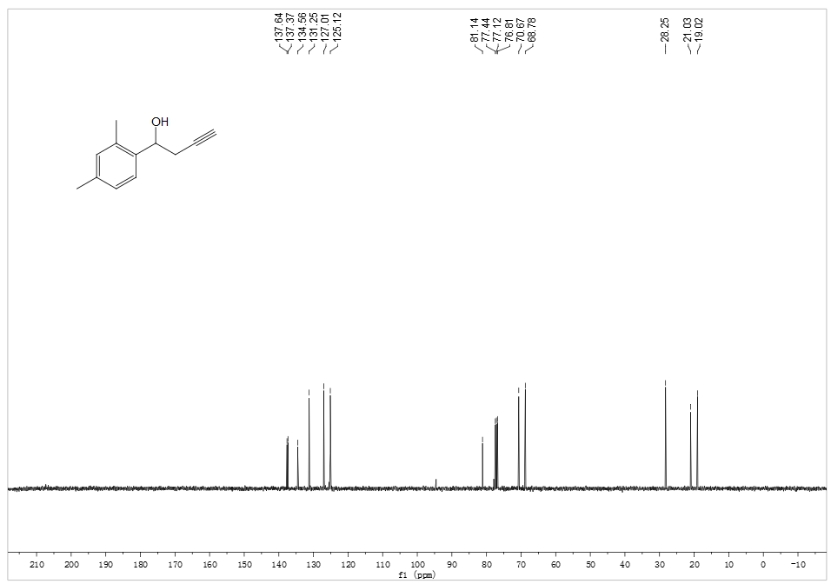


**^1^H NMR spectra of 1-(2,5-difluorophenyl)but-3-yn-1-ol (3n)**


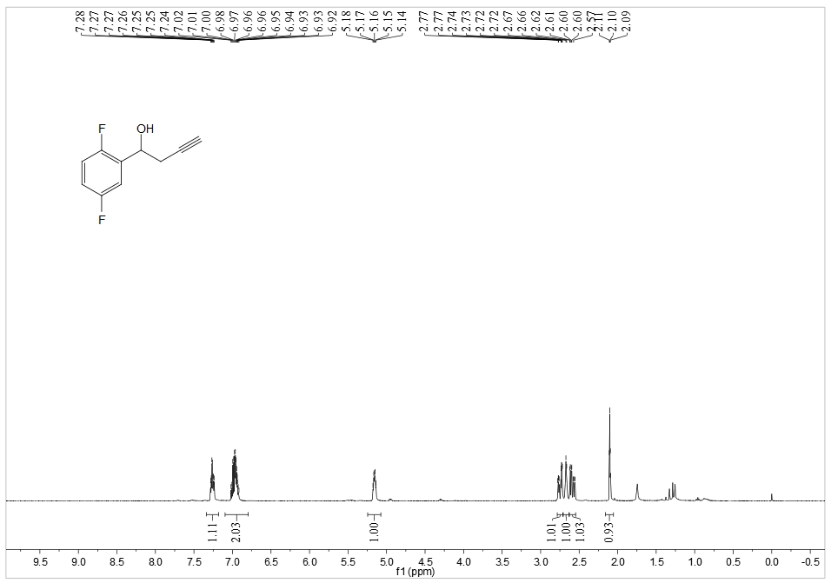


**^13^C NMR spectra of 1-(2,5-difluorophenyl)but-3-yn-1-ol (3n)** **
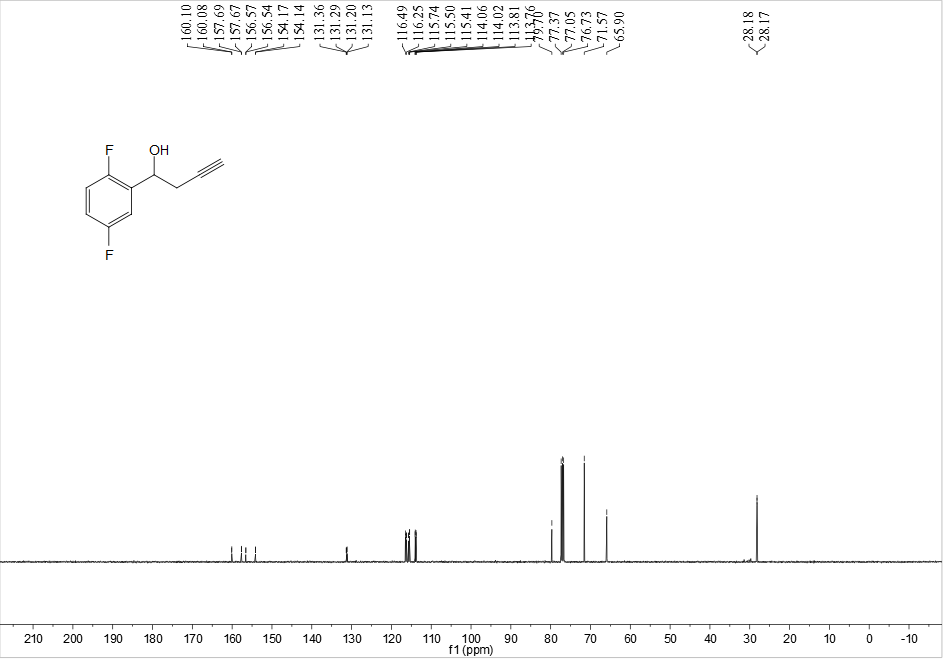
**

**^1^H NMR spectra of 1-(2,3-difluorophenyl)but-3-yn-1-ol (3o)**


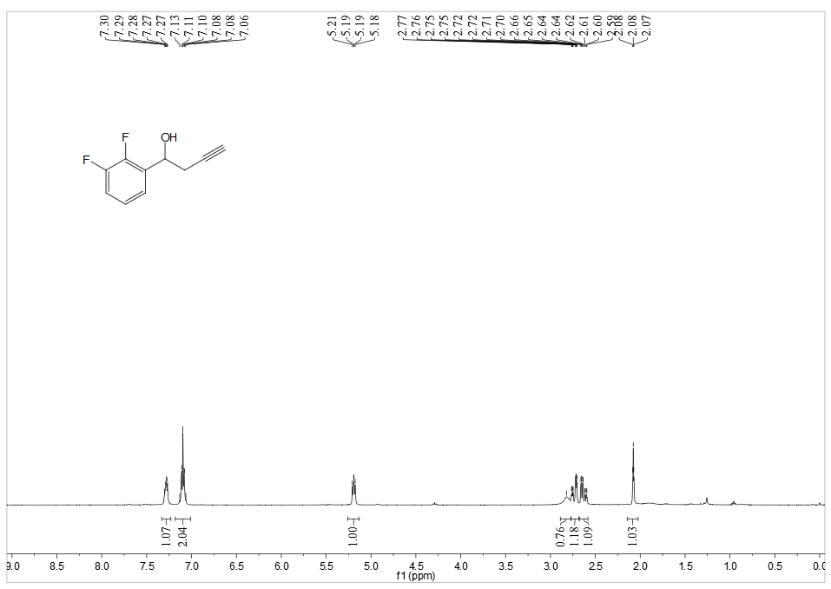


**^13^C NMR spectra of 1-(2,3-difluorophenyl)but-3-yn-1-ol (3o)**

**
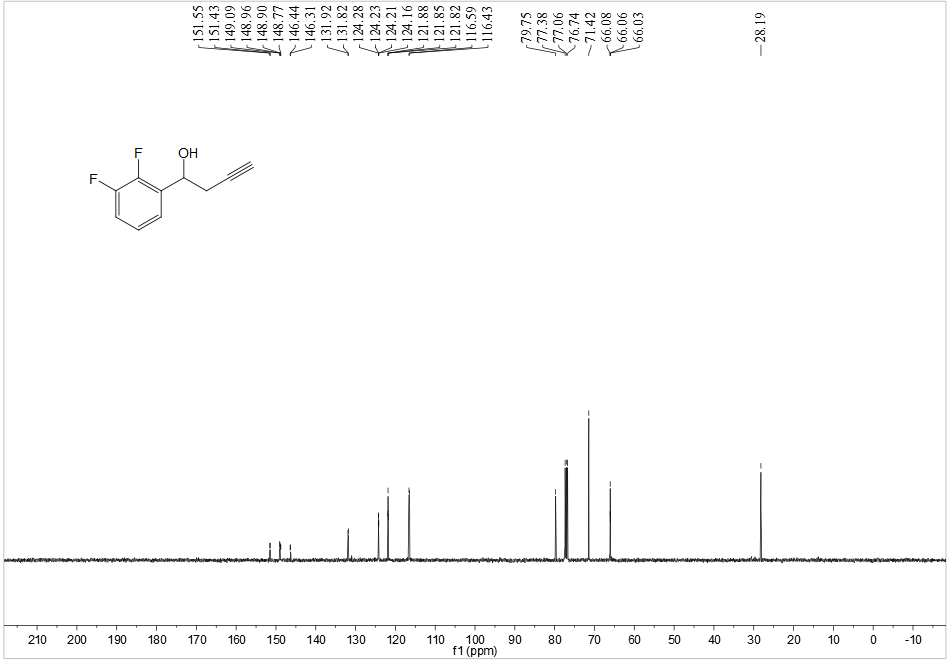
**

**^1^H NMR spectra of 1-(2,3-dimethylphenyl)but-3-yn-1-ol (3p)**


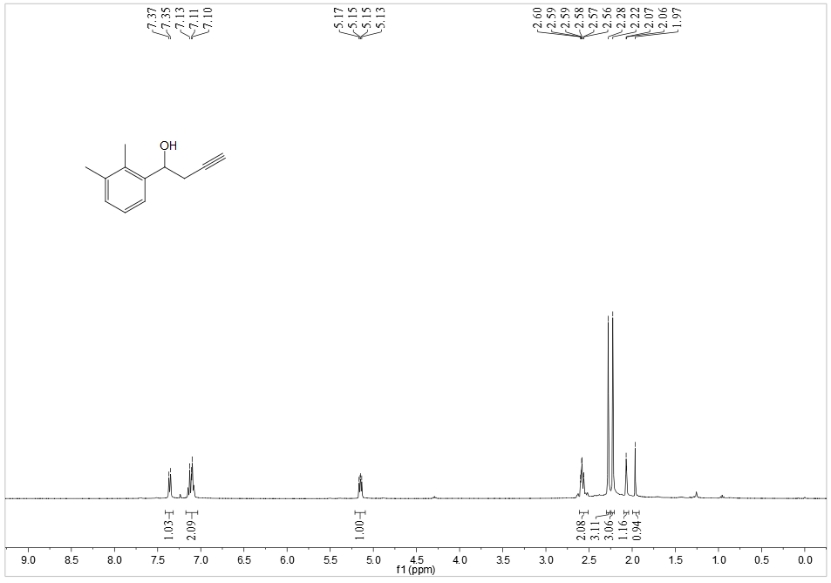


**^13^C NMR spectra of 1-(2,3-dimethylphenyl)but-3-yn-1-ol (3p)**


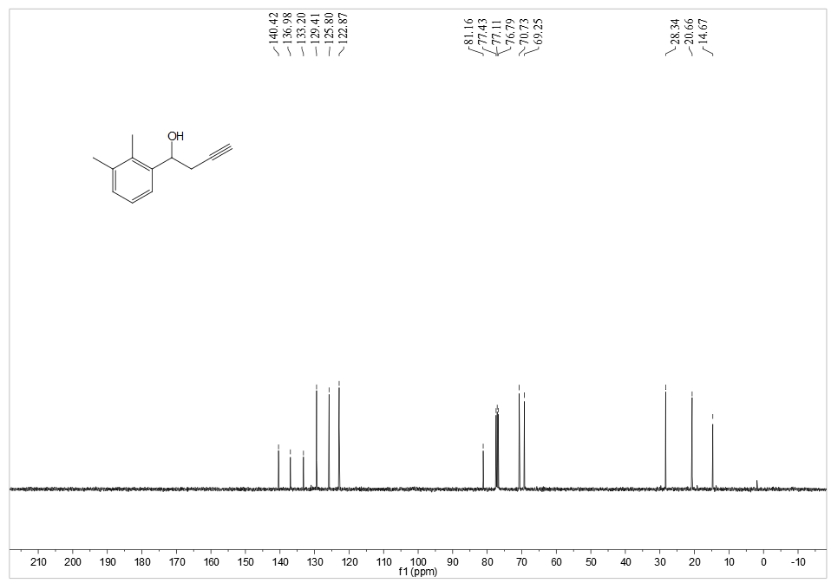


**^1^H NMR spectra of 1-(2-methoxy-4-methylphenyl)but-3-yn-1-ol (3q)**
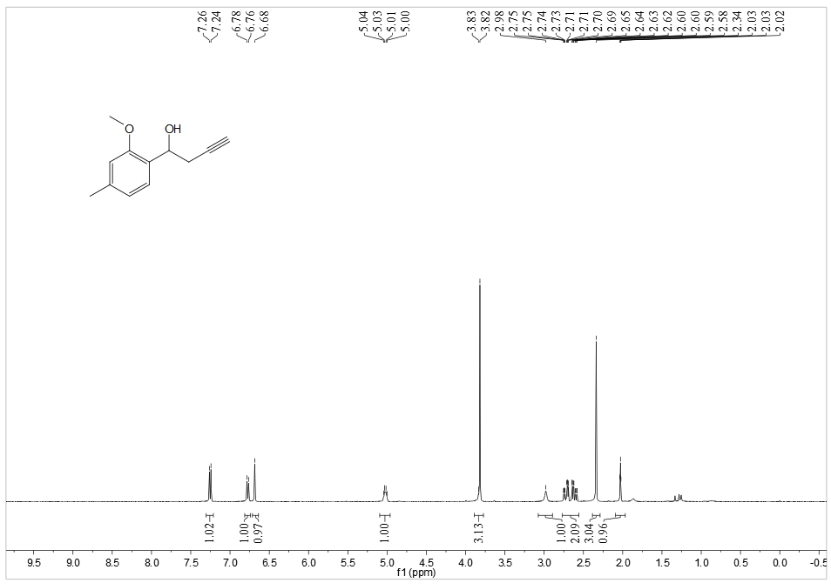


**^13^C NMR spectra of 1-(2-methoxy-4-methylphenyl)but-3-yn-1-ol (3q)**
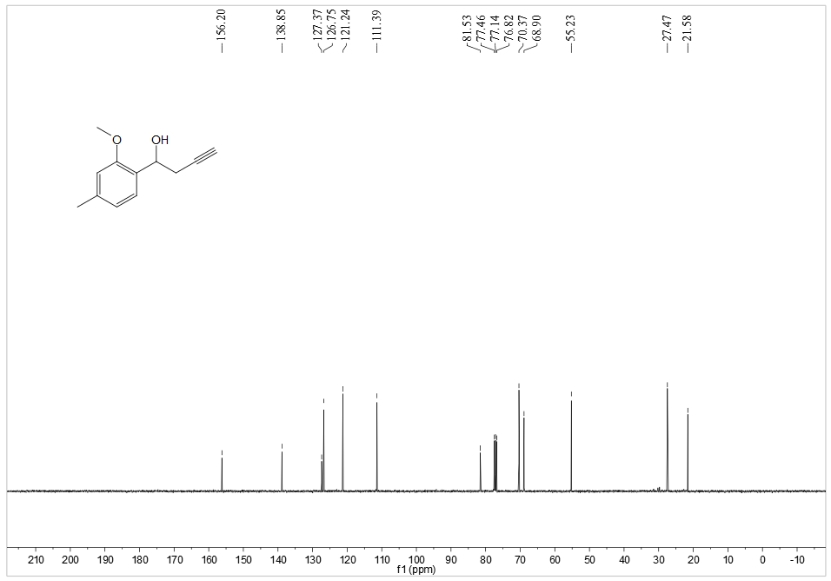


**^1^H NMR spectra of 1-(3-chloro-5-fluorophenyl)but-3-yn-1-ol (3r)**
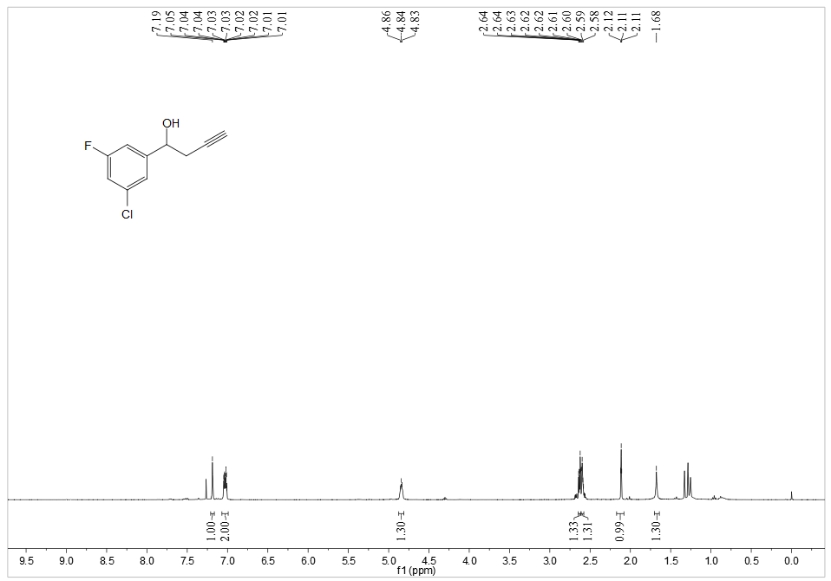


**^13^C NMR spectra of 1-(3-chloro-5-fluorophenyl)but-3-yn-1-ol (3r)**


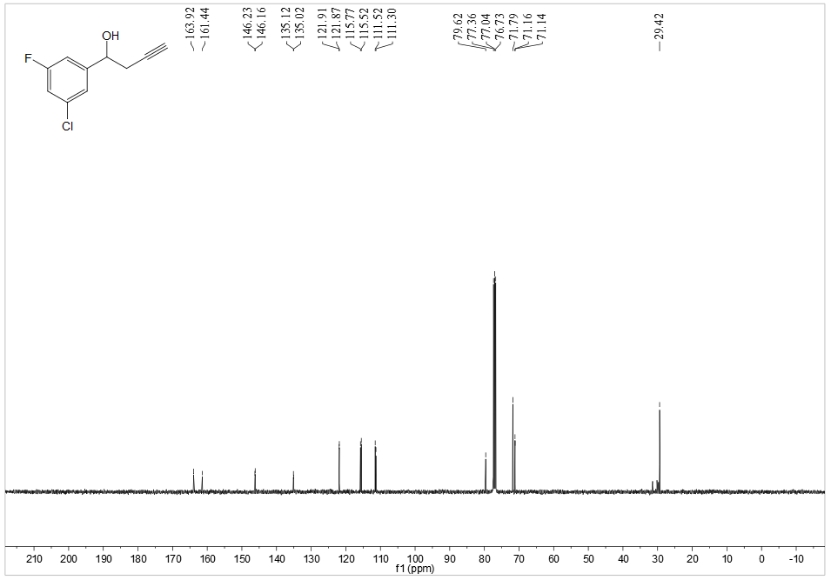


**^1^H NMR spectra of 1-(4-fluoro-3-methoxyphenyl)but-3-yn-1-ol (3s)**
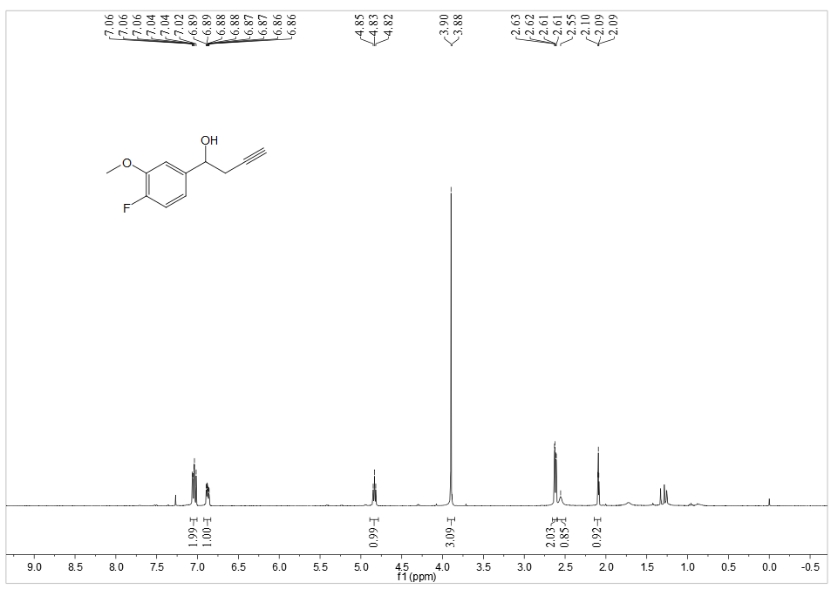


**^13^C NMR spectra of 1-(4-fluoro-3-methoxyphenyl)but-3-yn-1-ol (3s)**


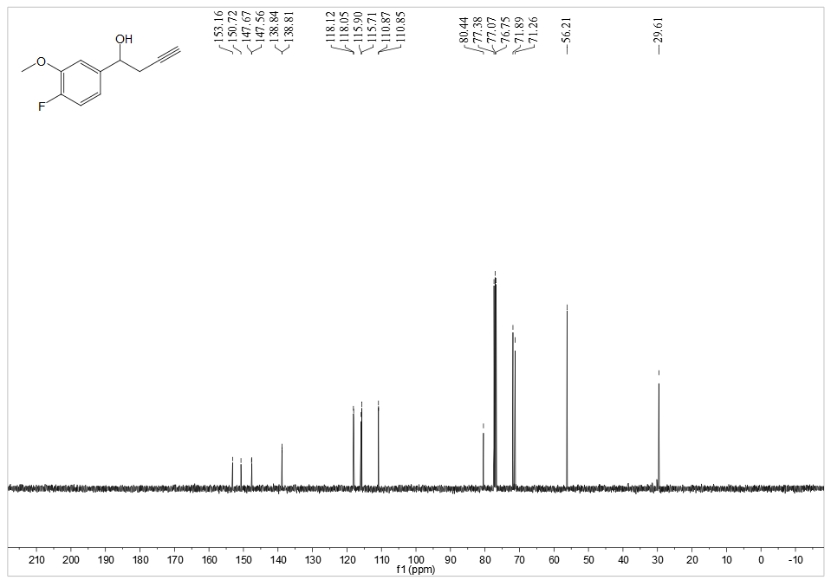


**^1^H NMR spectra of 1-(4-fluoro-3-methylphenyl)but-3-yn-1-ol (3t)**


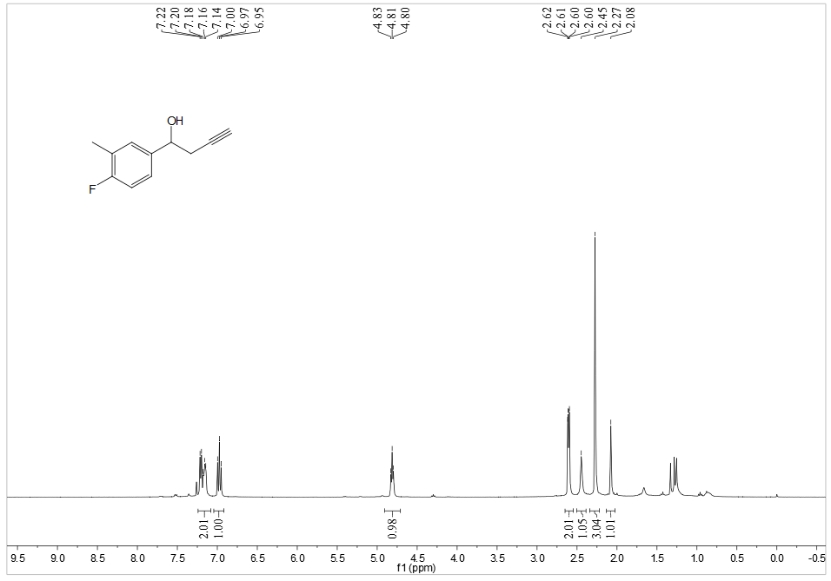


**^13^C NMR spectra of 1-(4-fluoro-3-methylphenyl)but-3-yn-1-ol (3t)**


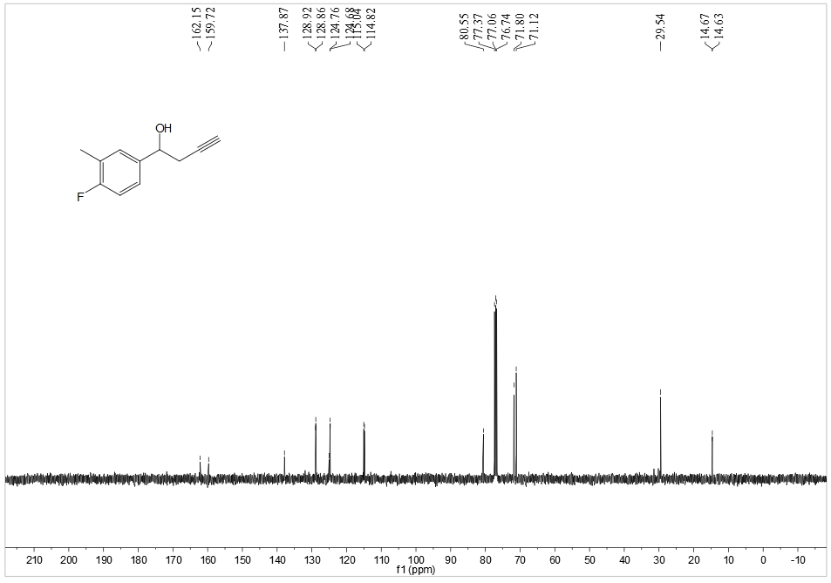


**^1^H NMR spectra of 1-(benzo[d][1,3]dioxol-4-yl)but-3-yn-1-ol (3u)**
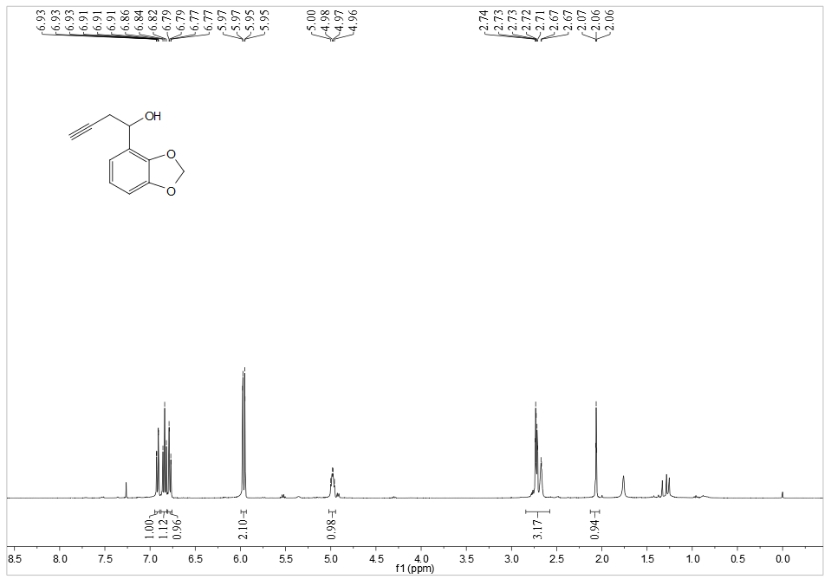


**^13^C NMR spectra of 1-(benzo[d][1,3]dioxol-4-yl)but-3-yn-1-ol (3u)**
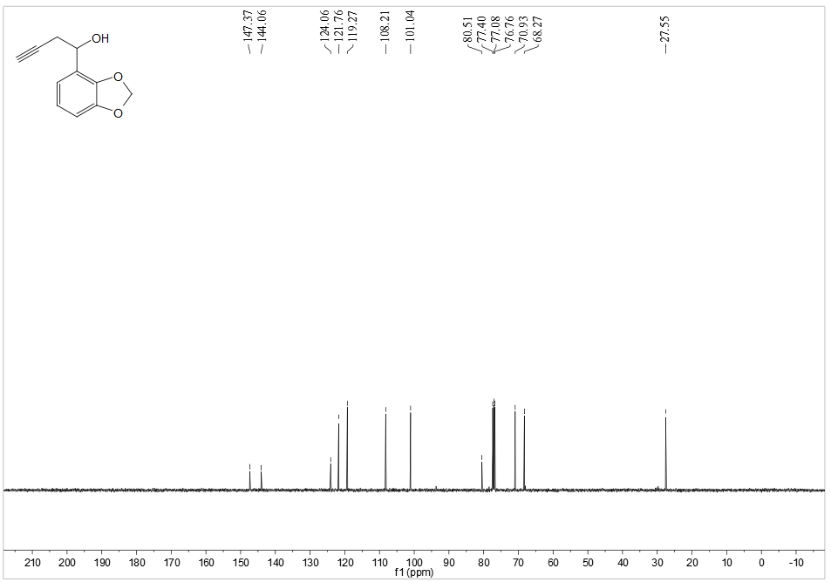


**^1^H NMR spectra of 1-(thiophen-2-yl)but-3-yn-1-ol (3v)**
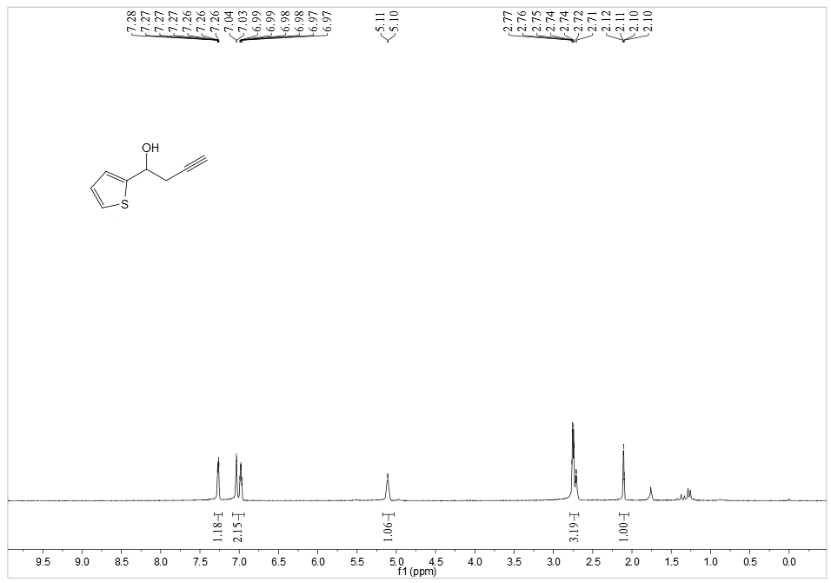


**^13^C NMR spectra of 1-(thiophen-2-yl)but-3-yn-1-ol (3v)**
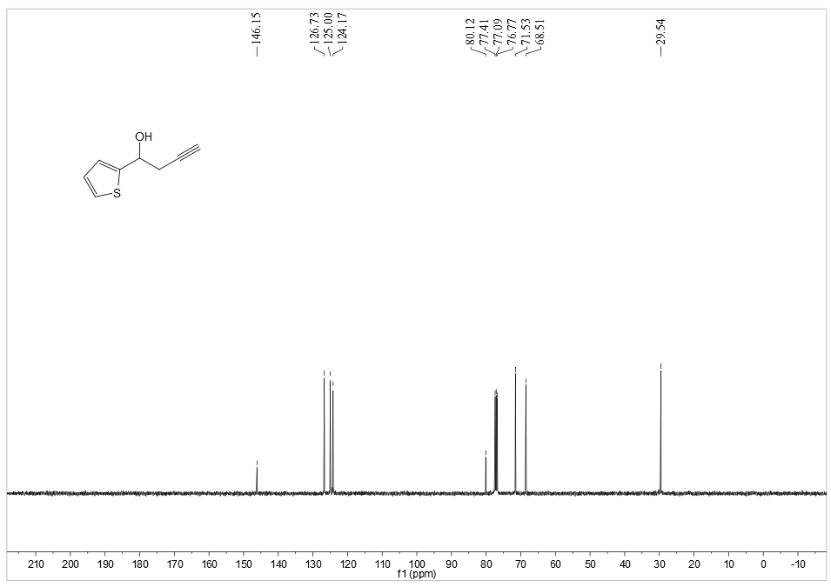


**^1^H NMR spectra of 1-(4-chloropyridin-2-yl)but-3-yn-1-ol (3w)**
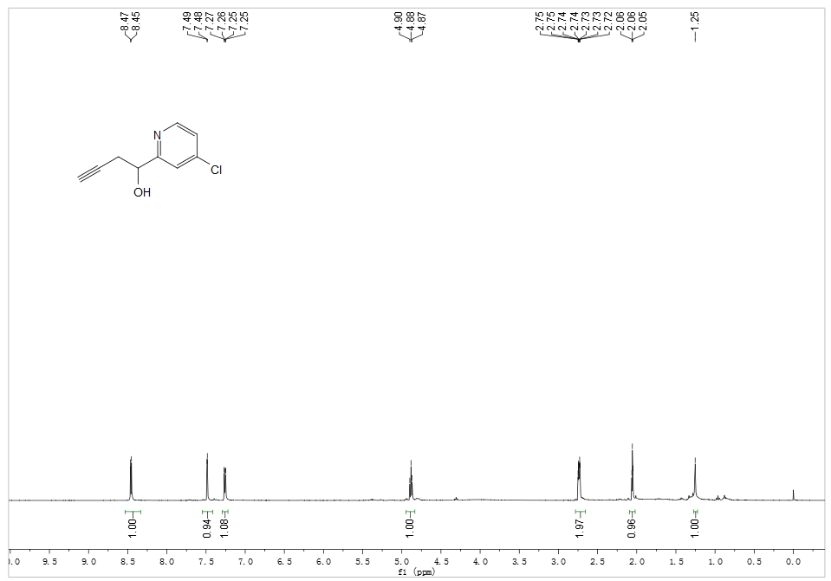


**^13^C NMR spectra of 1-(4-chloropyridin-2-yl)but-3-yn-1-ol (3w)**
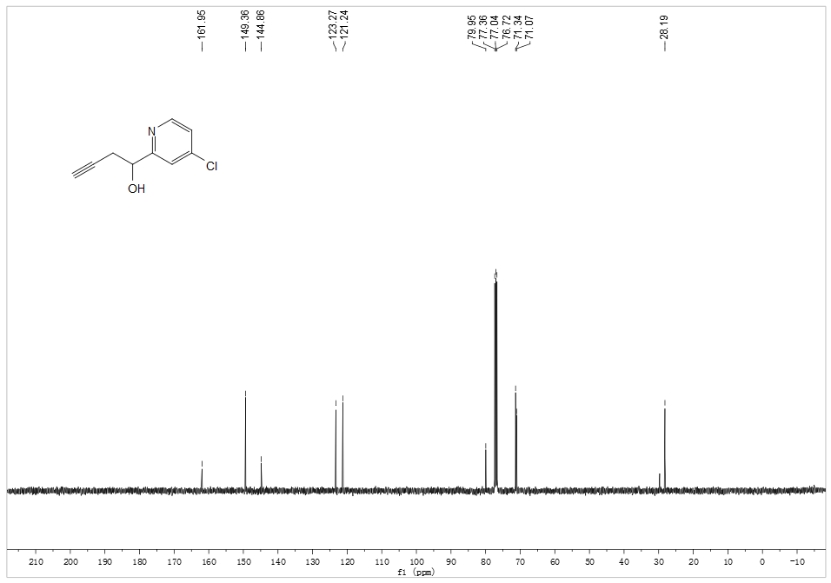


**^1^H NMR spectra of 1-(pyridin-3-yl)but-3-yn-1-ol (3x)**
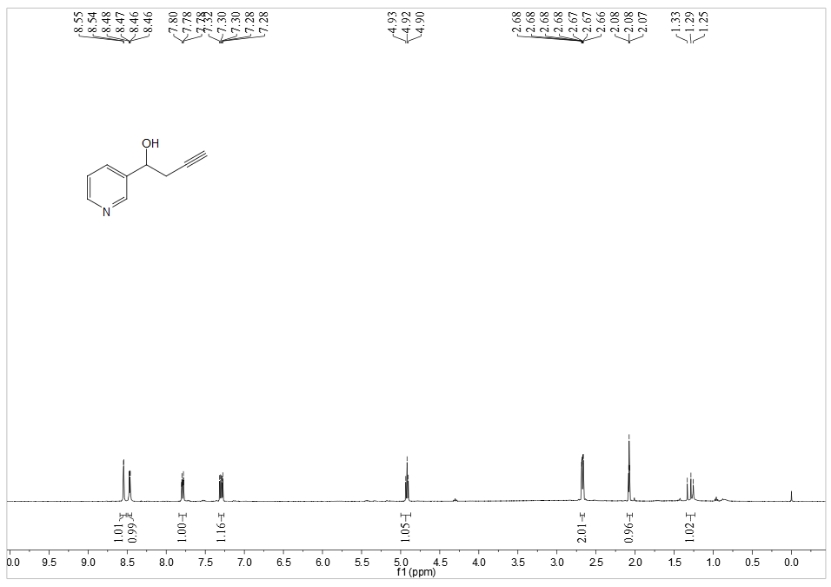


**^13^C NMR spectra of 1-(pyridin-3-yl)but-3-yn-1-ol (3x)**
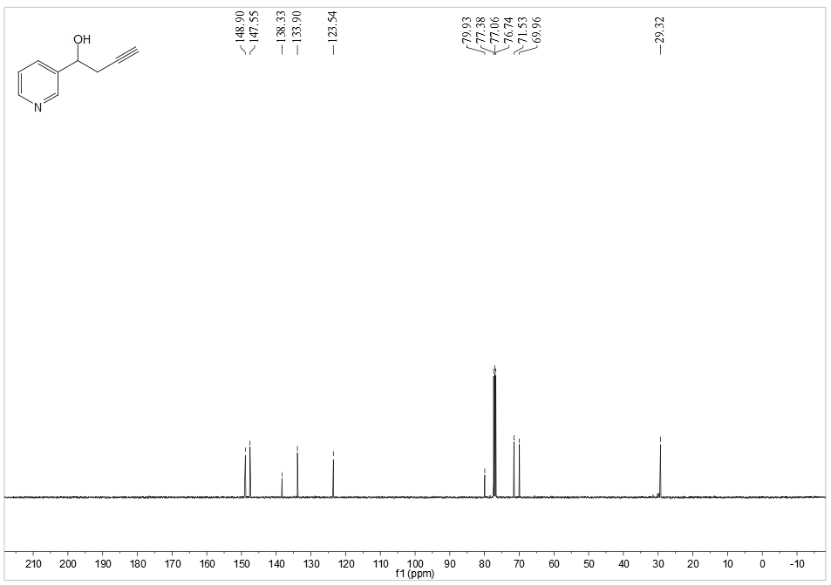


**^1^H NMR spectra of 1-(quinolin-2-yl)but-3-yn-1-ol (3y)**


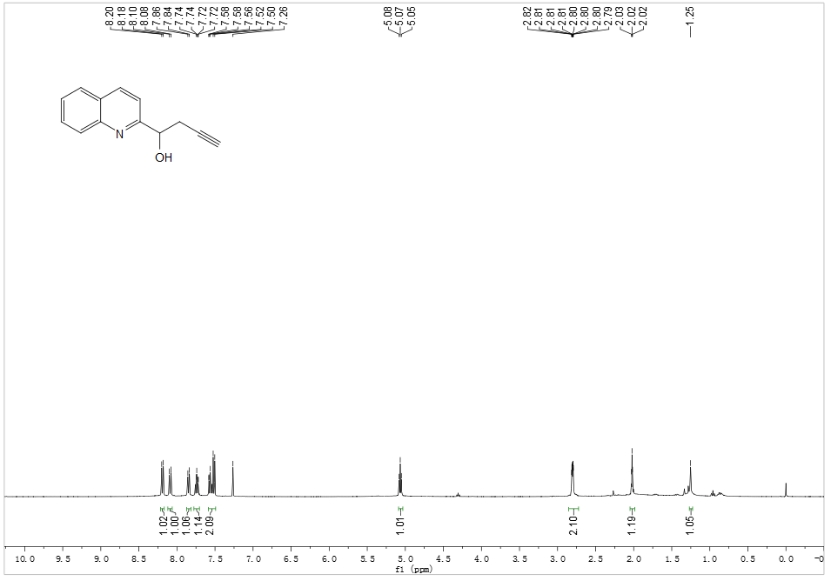


**^13^C NMR spectra of 1-(quinolin-2-yl)but-3-yn-1-ol (3y)**


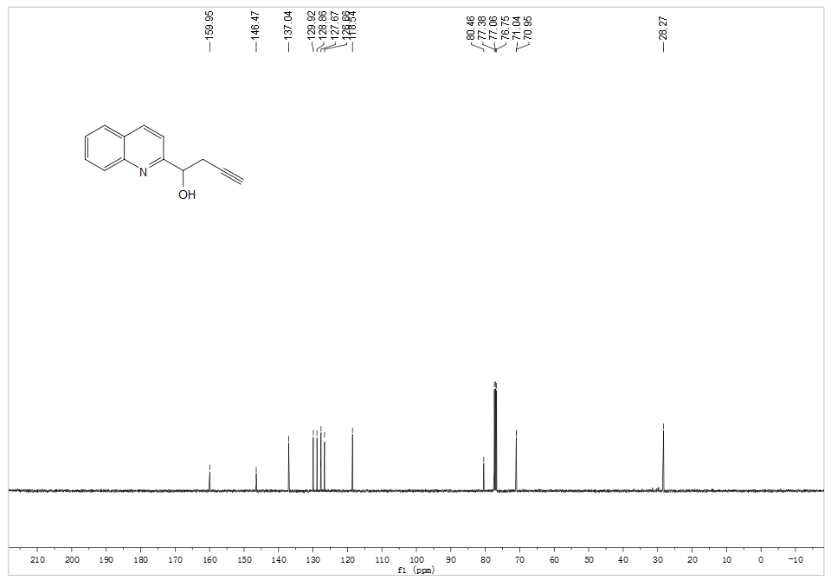


**1H NMR spectra of 1-(naphthalen-2-yl)but-3-yn-1-ol (3z)**


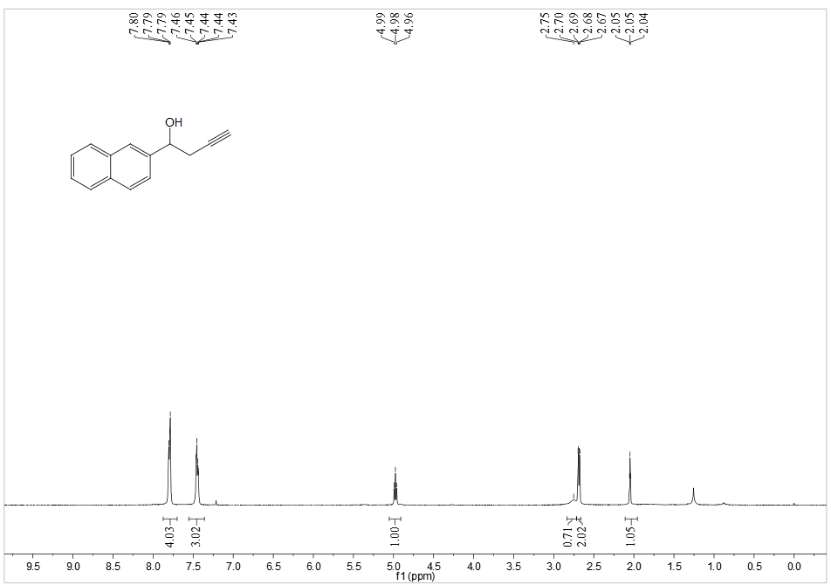


**13C NMR spectra of 1-(naphthalen-2-yl)but-3-yn-1-ol (3z)**


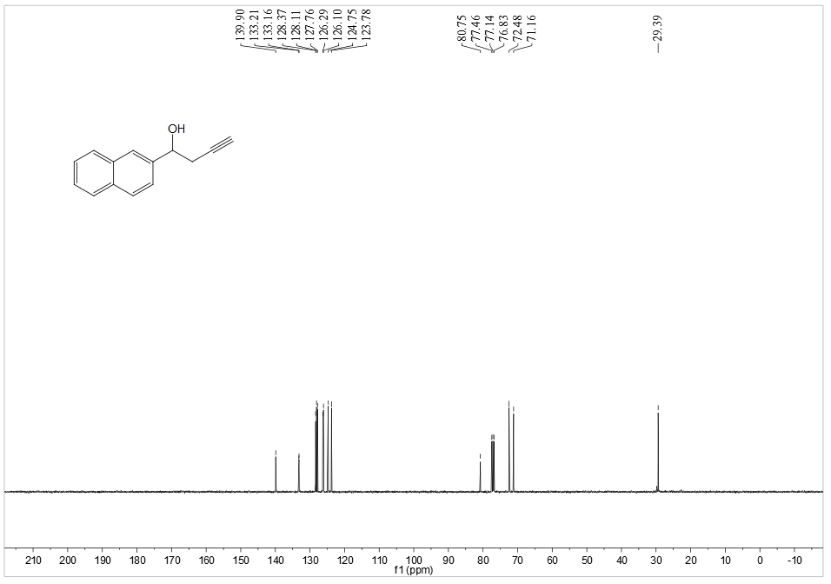


**1H NMR spectra of 1-(naphthalen-1-yl)but-3-yn-1-ol (3ab)**


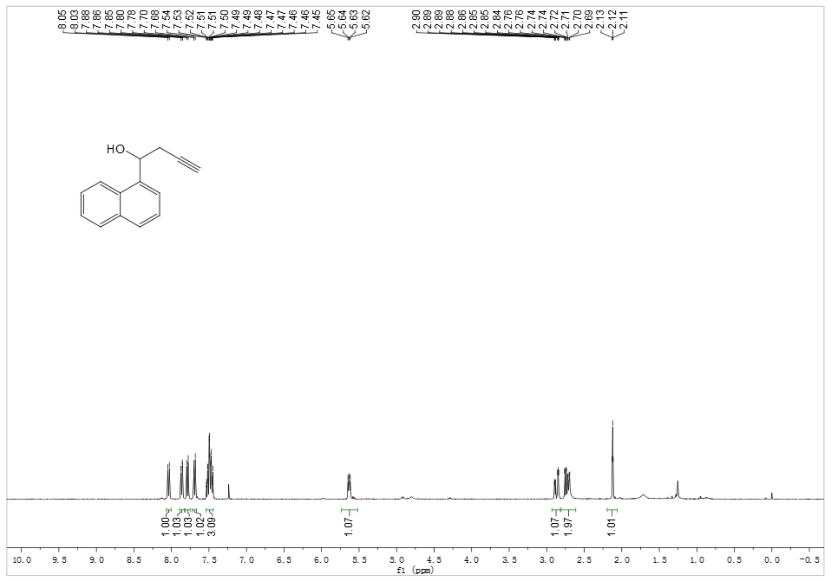


**13C NMR spectra of 1-(naphthalen-1-yl)but-3-yn-1-ol (3ab)**


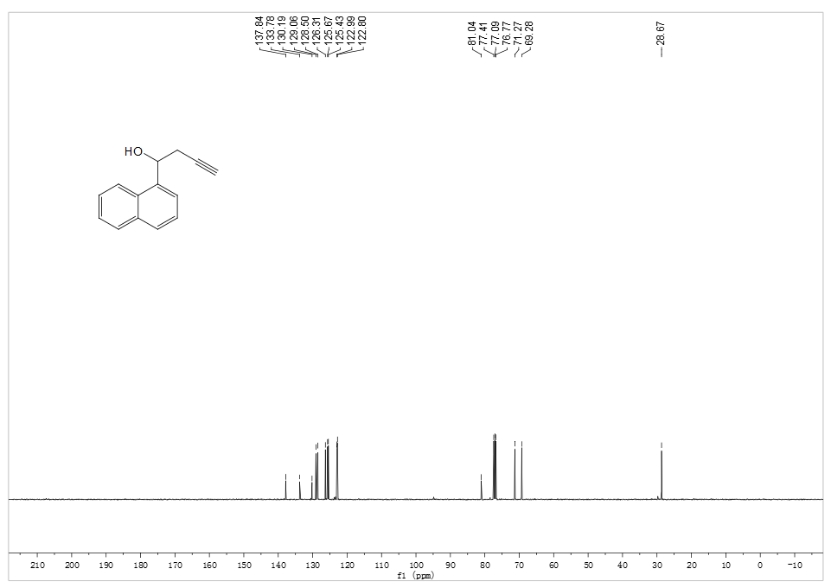


**1H NMR spectra of 1-(4-isopropylphenyl)-2-methyl-3λ^5^-buta-2,3-dien-1-ol (5a)**
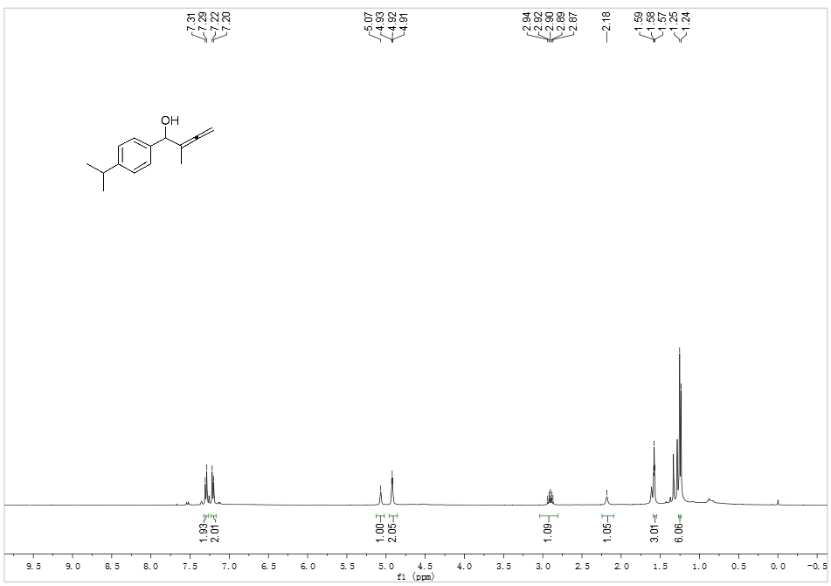


**13C NMR spectra of 1-(4-isopropylphenyl)-2-methyl-3λ^5^-buta-2,3-dien-1-ol (5a)**


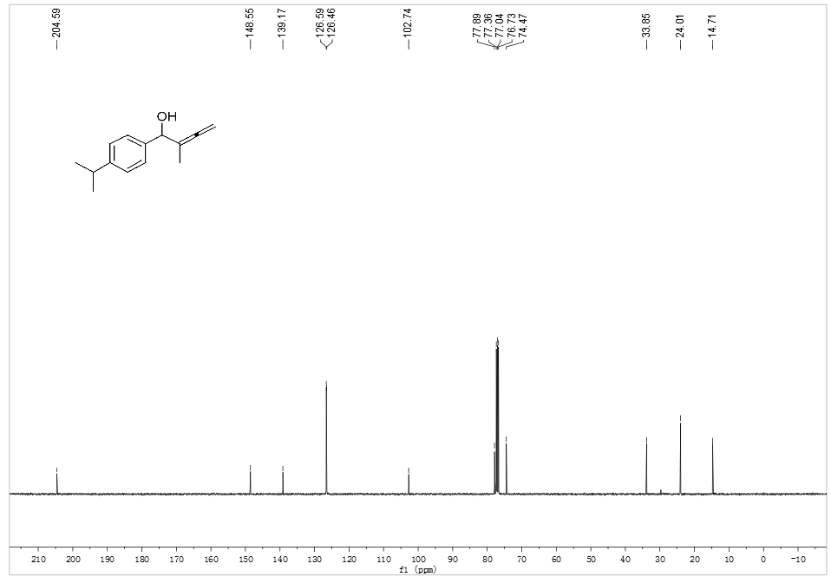


**1H NMR spectra of 2-methyl-1-(p-tolyl)-3λ^5^-buta-2,3-dien-1-ol (5b)**


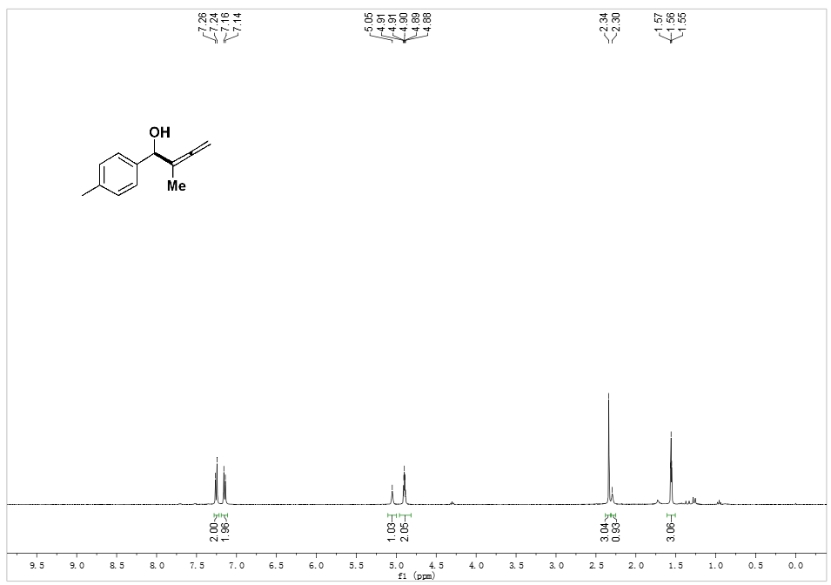


**13C NMR spectra of 2-methyl-1-(p-tolyl)-3λ^5^-buta-2,3-dien-1-ol (5b)**


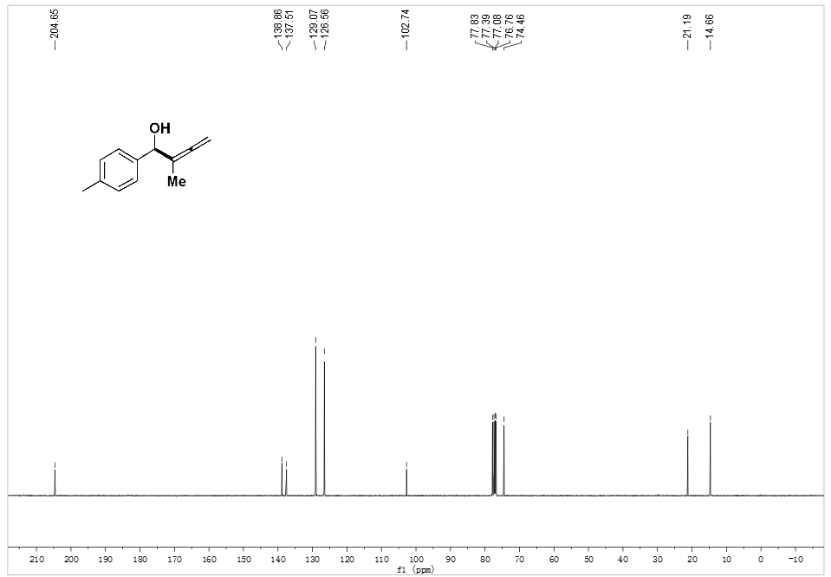


**1H NMR spectra of 1-(4-fluorophenyl)-2-methyl-3λ^5^-buta-2,3-dien-1-ol (5c)**


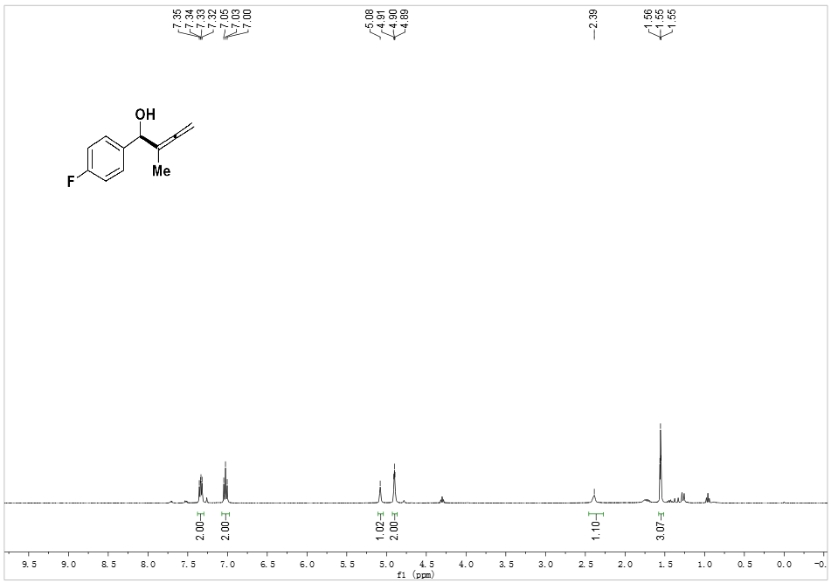


**13C NMR spectra of 1-(4-fluorophenyl)-2-methyl-3λ^5^-buta-2,3-dien-1-ol (5c)**


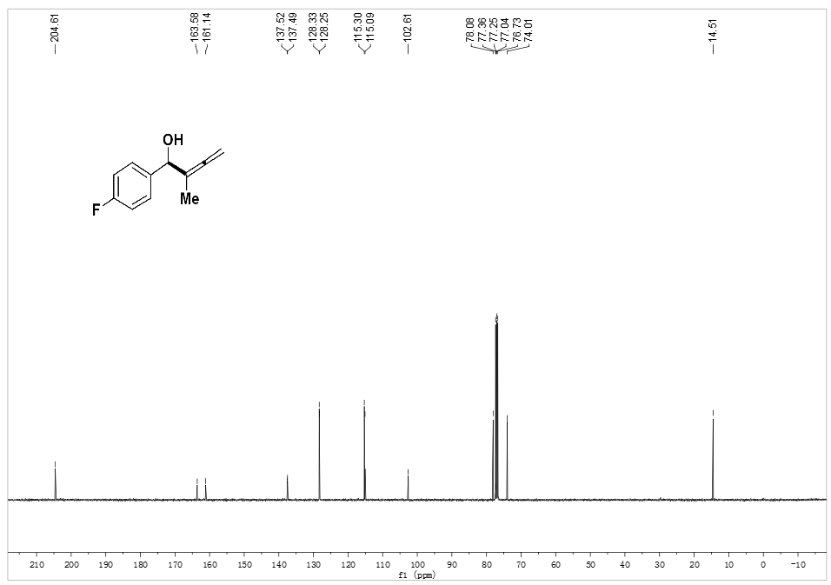


**1H NMR spectra of** **2-methyl-1-(thiophen-2-yl)-3λ^5^-buta-2,3-dien-1-ol (5d)**


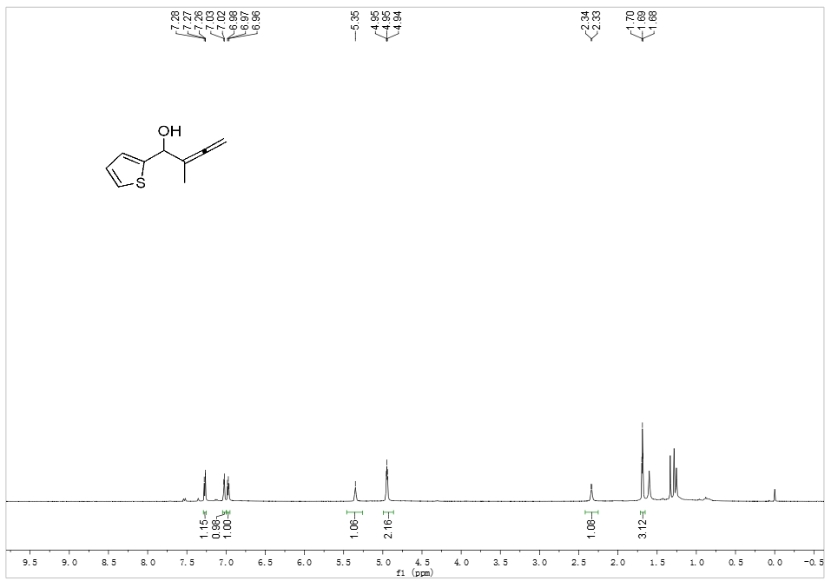


**13C NMR spectra of** **2-methyl-1-(thiophen-2-yl)-3λ^5^-buta-2,3-dien-1-ol (5d)**


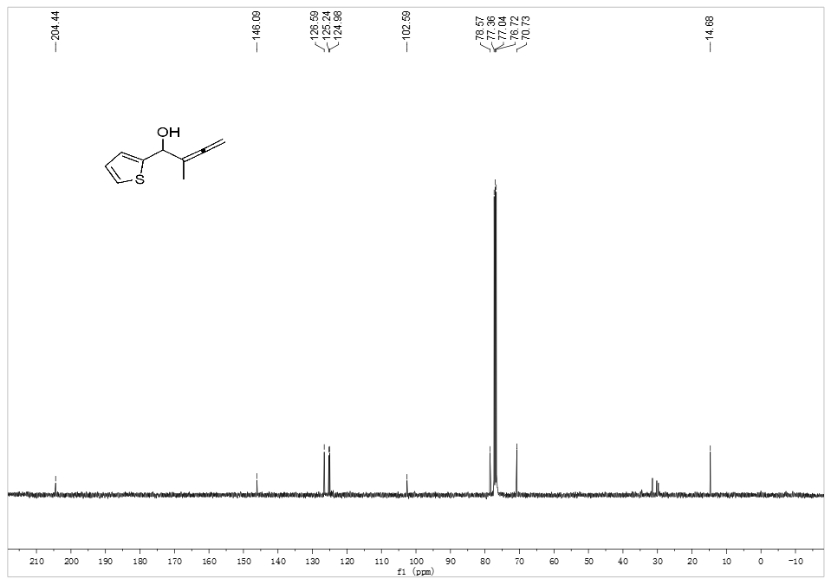

Supplement: Supplementary file 1 — Additional file 1. [file 13065_2022_803_MOESM1_ESM.doc]
